# Bone morphogenetic protein signaling: the pathway and its regulation

**DOI:** 10.1093/genetics/iyad200

**Published:** 2023-12-20

**Authors:** Takuya Akiyama, Laurel A Raftery, Kristi A Wharton

**Affiliations:** Department of Biology, Rich and Robin Porter Cancer Research Center, The Center for Genomic Advocacy, Indiana State University, Terre Haute, IN 47809, USA; School of Life Sciences, University of Nevada, 4505 S. Maryland Parkway, Las Vegas, NV 89154, USA; Department of Molecular Biology, Cell Biology, and Biochemistry, Carney Institute for Brain Science, Brown University, Providence, RI 02912, USA

**Keywords:** BMP signaling, Dpp, Gbb, DV patterning, NMJ, wing patterning, morphogen gradient, FlyBook, Tkv, Sax

## Abstract

In the mid-1960s, bone morphogenetic proteins (BMPs) were first identified in the extracts of bone to have the remarkable ability to induce heterotopic bone. When the *Drosophila* gene *decapentaplegic* (*dpp*) was first identified to share sequence similarity with mammalian BMP2/BMP4 in the late-1980s, it became clear that secreted BMP ligands can mediate processes other than bone formation. Following this discovery, collaborative efforts between *Drosophila* geneticists and mammalian biochemists made use of the strengths of their respective model systems to identify BMP signaling components and delineate the pathway. The ability to conduct genetic modifier screens in *Drosophila* with relative ease was critical in identifying the intracellular signal transducers for BMP signaling and the related transforming growth factor-beta/activin signaling pathway. Such screens also revealed a host of genes that encode other core signaling components and regulators of the pathway. In this review, we provide a historical account of this exciting time of gene discovery and discuss how the field has advanced over the past 30 years. We have learned that while the core BMP pathway is quite simple, composed of 3 components (ligand, receptor, and signal transducer), behind the versatility of this pathway lies multiple layers of regulation that ensures precise tissue-specific signaling output. We provide a sampling of these discoveries and highlight many questions that remain to be answered to fully understand the complexity of BMP signaling.

## Teaser

*Drosophila* genetics was instrumental in defining the BMP signaling pathway. In this FlyBook chapter, Akiyama, Raftery, and Wharton provide a historical account of the role that *Drosophila* research—especially genetic modifier screens—played in deciphering elements of BMP signaling and identifying its many functions. They cover more recent studies which demonstrate that the versatility of BMP signaling as a potent intercellular communication pathway lies in the diverse regulatory mechanisms that are critical to controlling signaling activity.

## Introduction

**Fig. 1.**
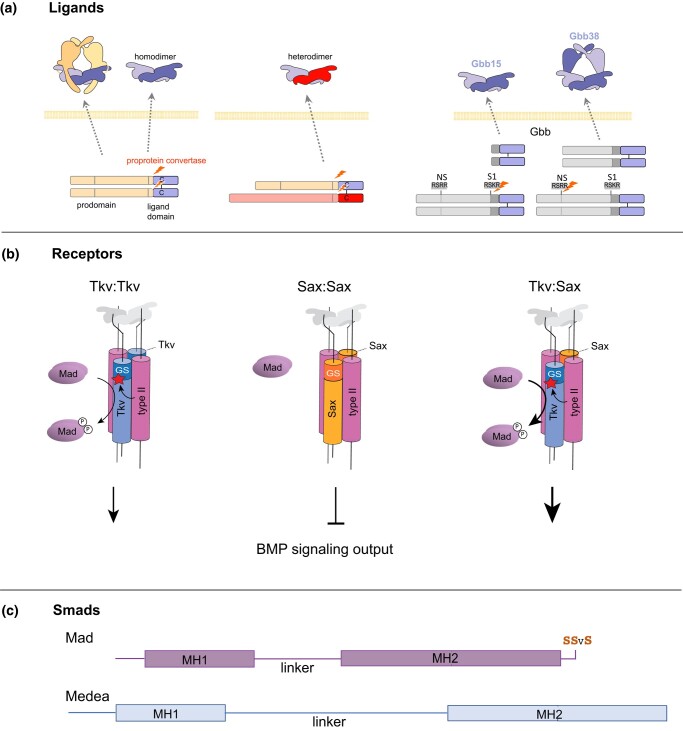
Core BMP signaling components. a) BMPs are synthesized as large proproteins that form dimers, linked by a disulfide in the C-terminal domain. The bioactive ligand (as a homodimer or heterodimer) consists of the C-terminal ligand domain and the associated prodomain, depending on the site of proteolytic cleavage by a proprotein convertase, such as furin. All cleaved products can be secreted (Anderson and Wharton 2017). Distinct ligand forms of Gbb (Gbb15 and Gbb38) have been observed *in vivo* and shown to have different functions. b) BMP type I receptors Tkv and Sax form tetrameric complexes with type II receptors (Punt and Wit). The constitutively active type II receptor kinase phosphorylates serine residues in the GS domain of the type I receptor to activate its kinase. The intracellular R-Smad, Mad, is thus phosphorylated. Receptor complexes containing Tkv are competent to signal, while those containing only Sax fail to propagate a signal by phosphorylating Mad despite binding ligand. c) Smads Mad (R-Smad) and Medea (co-Smad) share a primary structure of MH1 and MH2 domains separated by a linker. Mad is phosphorylated on its C-terminal serines by the type I receptor kinase, while at sites within the linker by other kinases (see Fig. 4).

The BMP pathway is a versatile cell signaling pathway that shows high conservation of its core components across 500 MY of metazoan evolution. The pathway is named for its ligands, BMPs or bone morphogenetic proteins. BMPs are peptides first identified in the bone extracts possessing the remarkable property of being able to induce heterotopic bone formation when injected subcutaneously in rats (Urist 1965). We now know that BMP signaling is not limited to the induction of bone but impacts a large number of developmental processes across the animal kingdom and whose disruption in humans is associated with many types of developmental abnormalities and disease (Wu and Hill 2009; Wang *et al*. 2014; Gomez-Puerto *et al*. 2019; Sconocchia and Sconocchia 2021). The pleiotropic nature of this signaling pathway initially came from studies primarily conducted in invertebrates. The transduction mechanism is responsible for receiving the extracellular signal to the nucleus where changes in transcription occur in response to BMP ligands. The active BMP ligand, a dimer of two ∼100aa peptides, is secreted and binds to the ectodomain of a heterotetrameric transmembrane receptor complex. The constitutively active type II serine/threonine (S/T) kinase phosphorylates and activates the type I S/T kinase upon ligand binding. The activated type I S/T kinase in turn activates a cytoplasmic transducer, the receptor-mediated Smad (R-Smad) protein via phosphorylation of discrete sites at the C-terminus, allowing it to accumulate in the nucleus where it regulates the transcriptional output of target genes (Fig. 1).

BMP signaling is used repeatedly throughout animal development, as well as during adult life. During evolution, the genes encoding BMPs have duplicated and diverged, such that over 20 BMPs exist forming 4 gene subfamilies (Newfeld *et al*. 1999; Bragdon *et al*. 2011; Zinski *et al*. 2018). Three BMPs, representing the BMP2/4 and BMP5/6/7/8a/8b subfamilies, are found in *Drosophila* (Table 1). The BMP family is the ancestral group of the transforming growth factor-beta (TGF-β) superfamily, which is comprised of related ligands: TGF-βs, activins, Growth/Differentiation Factor (GDFs), and BMPs, all sharing 7 conserved cystine residues, 6 of which are involved in intramolecular disulfide bonds to form a cystine knot, the structural element defining all members of the superfamily (reviewed in Hinck 2012, 2016; Goebel *et al*. 2019). The versatile nature of BMP signaling stems in part from the number of related BMP proteins comprising a gene family, coupled with their ability to form homodimeric and heterodimeric ligands and the presence of multiple receptors, as the 2 types of transmembrane receptors (type I and type II) responsible for mediating a BMP signal each display sequence conservation and form a gene family. The combinatorial assembly of both receptor and ligand components to generate an active signaling complex is varied. While the number of different ligand/receptor combinations, with different spatial and temporal expression patterns, provides for a high degree of diversity in signaling output, the past 20 years of research has revealed a multitude of molecular mechanisms that are layered on top of the variety of ligand/receptor interactions possible and act to regulate ligand, receptor, and signal transducer availability and activity.

**Table 1.** Drosophila and Human BMP and Activin signaling components.

BMP signaling components		Activin signaling components	
BMP ligand	Human ortholog	Activin ligand	Human ortholog
Dpp	BMP2/4	Myo	Myostatin, GDF11
Gbb	BMP5/6/7/8a/8b	Actβ	inhibinβA/βB
Scw	BMP5/6/7/8a/8b	Daw	inhibinβC/βE, TGF-β
		Mav	GDF15/Nodal/TGF-β3
Type I receptor		Type I receptor	
Tkv	ALK3/6	Babo	ALK4/5/7
Sax	ALK1/2		
Type II receptor		Type II receptor	
Punt	ACTRII/IIB	Punt	ACTRII/IIB
Wit	BMPRII	Wit	BMPRII
R-Smad		R-Smad	
Mad	Smad1/5/8	Smox/dSmad2	Smad2/3
Co-Smad		Co-Smad	
Medea	Smad4	Medea	Smad4
i-Smad			
Dad	Smad6/7		

In this review, we focus on the contribution of *Drosophila* genetic research to the elucidation of the core BMP/TGF-β signaling pathway, the identification of critical signaling components, and on the importance of regulating this potent and broadly used signal transduction pathway during development and in homeostasis in adult life. At the heart of the BMP/TGF-β pathway explosion in the late 1980–1990s was the willingness of *Drosophila* researchers and those studying mammalian BMPs and TGF-βs to collaborate. Together, progress was rapid, and the expertise that each group of scientists brought to the table was critical for the initial discoveries. Such collaborations have been equally important for subsequent studies which continue today, to understand how this potent signaling pathway is controlled in different cellular and developmental contexts and how its misregulation is the basis of tissue abnormality and disease.

Here, we provide a historical account that focuses on the discovery of the core BMP/TGF-β signaling components and the critical role that *Drosophila* genetics played in defining the pathway. In the next sections, we draw on studies performed in *Drosophila* in specific developmental contexts to highlight some of the molecular machineries that impose regulatory measures on the BMP pathway to modify its output in different cellular and developmental contexts. We will not provide a full review of the many roles of BMP signaling nor provide the details of its relationship to signaling by other TGF-β superfamily members, such as the activin and GDF subgroups. For such details, we refer readers to a number of outstanding reviews that cover such topics (Schmierer and Hill 2007; Miyazono *et al*. 2010; Shimmi and Newfeld 2013; Gaarenstroom and Hill 2014; Hamaratoglu *et al*. 2014; Grgurevic *et al*. 2016; Morikawa *et al*. 2016; Upadhyay *et al*. 2017; Gomez-Puerto *et al*. 2019). Flybase [flybase.org (Gramates *et al*. 2022)] is also an invaluable resource to which the *Drosophila* community contributes, where detailed information about each pathway component, its genetic and molecular properties, functions, and interactions with other factors can be found.

Our primary focus will be the contribution that *Drosophila* research made in breaking open the signaling field with the critical identification of core BMP signaling components and in helping to define the fundamental mechanisms for BMP signaling and its regulation. Collaborations between *Drosophila* researchers and those studying mammalian BMPs and TGF-βs were critical for these discoveries, as well as for subsequent studies that continue today, focused on understanding how this potent signaling pathway is controlled in many different cellular and developmental processes and how its misregulation is the basis of tissue abnormalities and disease. Here, we start with the discovery of the core BMP signaling components and then draw from various functional studies in *Drosophila* to demonstrate the different molecular machineries that impose regulatory measures on the core pathway to modify its output in different contexts. Certain contexts such as wing patterning and vein specification have been go-to systems for assessing functional relationships. An important feature of BMP (and TGF-β/Activin) signaling is that specific molecular mechanisms regulating signaling output have been found to be context dependent (Bragdon *et al*. 2011; Raftery and Umulis 2012; Morikawa *et al*. 2016; Upadhyay *et al*. 2017). We will not emphasize work that was initiated or discovered using *Drosophila* as a model system and will not provide a full review of BMP signaling and its many functions, nor will we provide details of the relationship between BMP signaling and signaling initiated by other TGF-β superfamily members, such as those that belong to the Activin subgroup. To aid the reader, Table 1 lists *Drosophila* BMP signaling components, their human orthologs, as well as Activin signaling components for completeness.

## Discovery of BMP core components

The pivotal role that *Drosophila* genetics played in delineating the BMP and TGF-β signaling pathways started with the realization that the *dpp* gene shared sequence similarities with the secreted mammalian BMP peptides, members of what has become known as the TGF-β superfamily (Padgett *et al*. 1987; Wozney *et al*. 1988). The stage was primed with the recent isolation of a new class of *dpp* alleles and the realization that *dpp* plays a critical role in dorsal/ventral (DV) patterning in the early embryo (Gelbart *et al*. 1985; Irish and Gelbart 1987; Wharton *et al*. 1993). At the same time, the landmark genetic screens of Christiane Nusslein-Volhard, Eric Weischaus, and Gerd Jürgens revealed that body patterning along the anterior/posterior (AP) and DV axes is governed by a discrete set of genes (Nusslein-Volhard *et al*. 1984; Anderson *et al*. 1985; Nusslein-Volhard *et al*. 1985; Wieschaus and Nusslein-Volhard 2016). It was soon recognized that genes that shared mutant phenotypes, such as defects in DV patterning, were likely to act in the same molecular pathway to achieve a common function, and this could be used as a criterion to search for components of a molecular pathway. Hand in hand with this classical genetic approach, the genome was being probed for genes, which shared conserved sequences with mammalian genes that encoded proteins newly identified via biochemical means to bind to members of the TGF-β/BMP family of ligands. This combined effort of geneticists, molecular biologists, and biochemists led to a rapid identification of the TGF-β/BMP signaling components. The rapid success was also due in large part to the willingness of *Drosophila* geneticists and mammalian biochemists to work together and leverage their respective expertise. With genetic interactions established between *dpp* alleles and those of the embryonic DV genes, coupled with modifier screens aimed at identifying second site mutations that enhance weak *dpp* phenotypes, the core BMP signaling pathway was quickly defined. This combination of genetic screens, molecular genetics, and biochemical studies in mammalian cells resulted in collaborations across multiple lab groups with the identity of core signaling components and their epistatic relationships defined in short order. Below, we first briefly summarize how each of the core signaling components, ligands, receptors, and Smads (Table 1, Fig. 1), were identified and then discuss the role that *Drosophila* genetics played in further defining the pathway and its regulation.

### The BMP ligands

The conservation of BMP signaling across animal phyla was brought home by the startling discovery of insect genes sharing homology with mammalian genes that encode bone-inducing peptides. Very soon, the early developmental roles for BMPs outside of the bone formation were appreciated based on results from functional studies in invertebrates, as well as in other vertebrates such as amphibians (Ramel and Hill 2012; Katagiri and Watabe 2016; De Robertis *et al*. 2017; Yan and Wang 2021). Like other members of the TGF-β superfamily, BMPs are secreted as dimers, both as homodimers and heterodimers (Bragdon *et al*. 2011). In *Drosophila*, 3 genes encode BMPs: *decapentaplegic* (*dpp*), *glass bottom boat* (*gbb*), and *screw* (*scw*).

***Dpp***: In 1937, *heldout* (*ho*), a mutation that alters adult wing posture was identified and mapped to 22F1-F3 on chromosome 2L (Novitski and Rifenburgh 1937). Subsequent studies of chromosomal inversions with breaks near *ho* demonstrated transvection and were shown to define a complex locus-designated *dpp* based on the fact that disruptions to the locus altered the development of the 15 imaginal discs that give rise to the “appendages” of the fly (Spencer *et al*. 1982; Gelbart *et al*. 1985). In 1987, the *dpp* locus was cloned and sequenced, and the C-terminal domain of the predicted Dpp protein sequence was shown to share sequence similarities with several mammalian proteins: TGF-β, Inhibin A/B, and MIS, members of the TGF-β gene family (Padgett *et al*. 1987). Shortly thereafter, vertebrate BMP2 and 4 were cloned and sequenced, revealing the conservation of their C-terminal sequences with the TGF-β gene family, as well as with Dpp (Wozney *et al*. 1988). In the same year, mutations in *dpp* were shown to be haploinsufficient with a requirement in DV patterning in the embryo (Irish and Gelbart 1987). Subsequently, these haploinsufficient mutations were mapped to the *dpp* coding region where they were shown to alter critical residues in the BMP ligand domain (St Johnston *et al*. 1990; Wharton *et al*. 1996), highlighting both the importance of Dpp as a BMP in the early specification of the DV axis, as well as dosage, i.e. that ligand concentration impacts the functional consequences of BMP signaling (Ferguson and Anderson 1992; Wharton *et al*. 1993, 1996). Recessive mutations outside of the *dpp* coding region that disrupt segments of the cis-regulatory regions resulted in mutant phenotypes reminiscent of those displayed by the larger chromosomal rearrangements exhibiting transvection leading to abnormalities in the development of the imaginal discs (Gelbart *et al*. 1985).

***Gbb***: A second *Drosophila* BMP gene at chromosomal position 60A on 2R was identified by degenerate PCR and shown to have sequence similarity with the vertebrate BMP5/6/7 subgroup (Wharton *et al*. 1991; Doctor *et al*. 1992). With the recovery of both null and hypomorphic alleles, the *60A* gene's role in cell fate specification during embryonic midgut and larval fat body development, as well as in wing imaginal disc patterning and ovary development, was evident (Khalsa *et al*. 1998; Wharton *et al*. 1999). Coincident with these studies a screen for genetic modifiers of *tkv* also identified lesions in 60A (Chen *et al*. 1998). Ultimately, the 60A gene was named *gbb* based on the ability to “see through” transparent mutant larvae, coupled with the fact that the letters g-b-b are a mirror image of d-p-p, capturing the observation that the anti-Gbb staining pattern in the wing imaginal disc is the inverse of the localized expression of *dpp* in a stripe of cells along the AP boundary (Khalsa *et al*. 1998; Wharton *et al*. 1999). In addition to roles in cell fate specification in the midgut and wing disc, *gbb* mutations have been found to affect tissue growth, metabolism, the maintenance of the germ cell niche, neuroblast proliferation, and synapse growth and function (Wharton *et al*. 1999; Kawase *et al*. 2004; Bangi and Wharton 2006a; Goold and Davis 2007; Ballard *et al*. 2010; Berke *et al*. 2013; Hong *et al*. 2016; Tian and Jiang 2017; Kanai *et al*. 2018; Hertenstein *et al*. 2021; see also Upadhyay *et al*. 2017).

***Scw***: The third *Drosophila* BMP gene, *scw*, was initially identified in a screen for genes acting in early embryonic pattern formation (Nusslein-Volhard *et al*. 1984). Its name arises from an embryonic lethal phenotype that results from defects in DV patterning. Upon gene cloning and sequencing, *scw* was shown to share amino acid sequence similarities with Gbb, Dpp, and other members of the vertebrate BMP family (Arora *et al*. 1994). Phylogenetic studies have since determined in the dipteran lineage that *scw* results from a duplication of *gbb* followed by divergence (Newfeld *et al*. 1999; Fritsch *et al*. 2010). The molecular screen using degenerate PCR to identify *gbb* failed to recover *scw* because of very low sequence conservation in the region of the locus covered by one of the primer sets (Wharton *et al*. 1991). *scw* expression is limited to the embryonic stage where it collaborates with *dpp* in defining distinct levels of signaling output necessary for patterning different cell fates within the dorsal ectoderm (Nguyen *et al*. 1998; Eldar *et al*. 2002; Shimmi, Umulis, *et al*. 2005; Wang and Ferguson 2005).

### The BMP receptors

BMP receptors consist of 2 forms, type I and type II, transmembrane, S/T kinase receptors that form a heterotetrameric signaling complex made up of 2 type I and 2 type II receptors (Yamashita *et al*. 1996). The first identification of signaling receptors for the TGF-β family of ligands was not made in *Drosophila* and was not based on specificity to BMP ligands. TGF-β binding proteins were identified by affinity-labeling assays in mammalian cells whereby 2 glycoproteins of 53kd and 75kd were identified as required for the growth response induced by TGF-β treatment (reviewed in Massagué 1992). These type I and type II receptors were recognized to constitute a family of related transmembrane S/T kinases conserved in *Caenorhabditis elegans* (Daf-1; Georgi *et al*. 1990) and *Drosophila* (Childs *et al*. 1993). Soon multiple members of type I receptor, as well as type II receptors, were identified in the early to mid-1990s representing subgroups with varying binding affinities for specific classes of TGF-β superfamily members, i.e. the BMPs, TGF-βs, and Activins (reviewed in Massagué *et al*. 1994; Miyazono *et al*. 2010). BMPs bind the type I receptor that recruits the constitutively active type II receptor (Wrana *et al*. 1994). The type II kinase phosphorylates the type I juxtamembrane GS domain, activating the type I kinase. The *Drosophila* BMP type I receptors are encoded by *tkv* (*thick veins*) and *sax* (*saxophone*), with *put* (*punt*) and *wit* (*wishful thinking*) encoding type II receptors.

***Type I receptors Tkv and Sax***: Both *tkv* and *sax* were originally identified based on mutant phenotypes prior to their subsequent identity as genes encoding BMP type I receptors. *thick veins* was first identified by Edith Wallace as mutations in the gene that produced thickened wing veins (Lindsley 1992). The *tkv* gene was mapped to 25D6-7 based on a report that one breakpoint of the B137 T(Y;2) translocation failed to complement *tkv* (Ashburner *et al*. 1980). Its subsequent identification in the (Nusslein-Volhard *et al*. 1984) screen for embryonic patterning genes (Jurgens *et al*. 1984), and studies of *tkv*'s role in embryonic patterning (Szidonya and Reuter 1988; Terracol and Lengyel 1994), tied *tkv* to specification of the DV axis, a process requiring other BMP signaling components. Very quickly, cloning and sequencing by at least 4 labs showed that *tkv* encodes a type I receptor (Brummel *et al*. 1994; Nellen *et al*. 1994; Okano *et al*. 1994; Penton *et al*. 1994).

*sax* was identified as a recessive female sterile with disrupted patterning of mutant embryos appearing as a twisted, J-shape, like a saxophone (Schupbach and Wieschaus 1989). The demonstration that *sax* alleles enhance the *dpp* loss of function DV patterning defects (Twombly *et al*. 1992) fueled the research that demonstrated that *sax* encoded another type I receptor (Brummel *et al*. 1994; Nellen *et al*. 1994; Okano *et al*. 1994; Penton *et al*. 1994; Xie *et al*. 1994).

***Type II receptors Punt and Wit***: *punt* (*put*) was isolated as a zygotic lethal on the third chromosome with abnormal embryonic DV patterning that resembles a flat boat due to its “dorsal-open phenotype” (Jurgens *et al*. 1984), similar to the embryonic *tkv* phenotype (Nusslein-Volhard *et al*. 1984; Terracol and Lengyel 1994). In an attempt to identify receptor genes based on conserved vertebrate type II receptor sequences, low stringency screening of genomic libraries highlighted the cytologic position 88C3-E3, the genomic region to which *punt* had originally been mapped (Childs *et al*. 1993; Wrana *et al*. 1994; Letsou *et al*. 1995; Ruberte *et al*. 1995). Again, the shared involvement in DV patterning in *Drosophila* coupled with the realization that genes encoding components of this pathway were conserved in mammals sped up the discovery of all key core signaling components. Cloning and sequencing confirmed the presence of Punt, the *Drosophila* activin-related type II receptor. Wit, a second type II receptor showing the highest sequence similarity to mammalian BMPRII, was first revealed by low-stringency sequence probes (Marques *et al*. 1996; Aberle *et al*. 2002). Mutations in *wit,* or *wishful thinking*, did not affect DV patterning but instead exhibited roles in synaptic growth and function, as well as in eggshell patterning and neuronal remodeling (Aberle *et al*. 2002; Marqués *et al*. 2002; Zheng *et al*. 2003; Yakoby, Bristow, *et al*. 2008, Yakoby, Lembong, *et al*. 2008; Marmion *et al*. 2013).

### The Smad signal transducers

When it was recognized that the C-terminal domain of Dpp shared sequence similarities with what became known as the TGF-β superfamily of secreted signaling ligands, the signal transduction pathway for this family was unknown. Screens for genetic modifiers of *dpp* provided the key to intracellular regulation, through the discovery of the founding member of the Smad family, *Mothers against dpp* (*Mad*). We now know that Smads transduce the intracellular signal from the cell surface receptors to the nucleus, where they regulate the expression of BMP-responsive genes.

Incidental observations from *Drosophila* geneticists suggested that some components of the Dpp signaling pathway might be maternally loaded into the zygote because the genetic background of the mother influenced the haploinsufficiency of several *dpp^hr^* alleles. Inspired by genetic enhancer screens performed by Simon *et al*. (1991) and Hafen *et al*. (1993), members of the Gelbart lab performed 2 pilot screens, a maternal-effect, as well as a zygotic screen for dominant enhancers of the *dpp* embryonic phenotype (Raftery *et al*. 1995) taking advantage of the partial haplolethality of *dpp^hr4^* (Irish and Gelbart 1987; Wharton *et al*. 1993). The maternal-effect screen for dominant enhancers of *dpp^hr4^* yielded 4 alleles of a locus on 2L and 3 alleles of a locus on 3R, which were named respectively *Mothers against dpp* (*Mad*) and *Medea* (*Med*; Raftery *et al*. 1995). Both were named for the dominant maternal effect of genetic interaction with *dpp^hr^* alleles, leading to the lethality of *dpp^hr^* heterozygous embryos. *Mad* refers to the social action organization “Mothers Against Drunk Driving” (MADD). *Medea* refers to the vindictive figure from ancient Greek mythology as depicted by Euripides (Euripedes 431 Bce), who exerts vengeance on her husband through the deaths of their children. Both *Mad* and *Medea* were reported to be predominantly early pupal lethal with mutant larvae displaying small discs and other phenotypes reminiscent of specific *dpp* allelic combinations (Raftery *et al*. 1995; Sekelsky *et al*. 1995; Wisotzkey *et al*. 1998).

***Mothers against Dpp (Mad):*** In an unrelated screen aimed at identifying the disruptors of *dpp* transvection, the founding *Mad^1^* allele was recovered (Sekelsky *et al*. 1995). *Mad^1^*, an EMS-induced mutation, exhibited a stronger maternal effect on *dpp*^hr^/+ progeny than a deficiency. Three other EMS-generated *Mad* alleles were semiviable in heteroallelic combinations, *Mad^11^* in combination with either *Mad^5^* or *Mad^6^*; the surviving adults had shortened legs, with loss of the most distal tarsal segments (Sekelsky *et al*. 1995). Flybase curators subsequently deduced that a previously reported gene, *apang*, most likely was the same locus as *Mad*. *apang* mapped to the same vicinity as *Mad* by meiotic recombination (Shekaran and Sharma 1983; Lindsley 1992) and showed a distal leg phenotype similar to viable *Mad* mutant allelic combinations. The open nature of the *Drosophila* research community facilitated the identification of additional alleles contributed by other labs. Assays for potential genetic null alleles of Mad were based on their allelic strength as dominant maternal-effect modifiers of *dpp^hr^* alleles (Sekelsky *et al*. 1995), identifying *Mad^7^*, *Mad^10^*, and *Mad^12^*. In parallel to the Gelbart lab screens, the Mlodzik lab identified a P element insertion in *Mad*, *l(2)k5807*, in a screen for dominant enhancers of the roughened eye phenotype associated with overexpression of Scabrous in the developing eye (Wiersdorff *et al*. 1996). This allele was renamed *Mad^B1^*.

*Mad* was cloned and sequenced, revealing a protein with no known structural domains, but with homology to sequences in the *C. elegans* genome that were ultimately associated with the *C. elegans small* (*sma*) genes (Savage *et al*. 1996). The novel sequence of Mad gave little clue to its function, but alignment with numerous vertebrate cDNAs highlighted 2 substantial domains of homology: Mad Homology Domain 1 (MH1) and Mad Homology Domain 2 (MH2; Fig. 1, reviewed in Raftery and Sutherland 1999). Heterozygosity for various *Mad* alleles could partially suppress the wing or eye phenotype resulting from the expression of constitutively active type I receptor, Tkv [both Tkv^Q199D^ (Hoodless *et al*. 1996) and Tkv^Q253D^ (Wiersdorff *et al*. 1996)]. Rapid progress on human Mad homologs demonstrated that they were phosphorylated at C-terminal serines by either a TGF-β type I receptor or a BMP type I receptor (Macías-Silva *et al*. 1996; Kretzschmar *et al*. 1997; Macías-Silva *et al*. 1998; Massagué 2000; Shi 2001). In the case of the human Mad homolog, Smad1, phosphorylation was blocked when Smad1 was mutated to carry the analogous molecular lesion seen in *Mad^10^* (G409S in Mad-PA, G479S in Mad-PB; Hoodless *et al*. 1996). The lesion associated with another null allele *Mad^12^* leads to a C-terminally truncated protein (Q147stop in Mad-PA) within the conserved MH2 domain. Many studies have since used *Mad^12^* homozygous cells to test whether BMP signaling has a causative role in specific physiological or development events. Given the sequence similarity between the *Drosophila Mad* and *C. elegans sma* genes, the nomenclature was consolidated to call this family of BMP and TGF-β signal transducing proteins, the Smads (Derynck *et al*. 1996).

***Medea (Med)***: In addition to the 3 alleles of *Medea* isolated in the maternal enhancer of *dpp^hr4^* screen (Raftery and Sutherland 1999), 2 alleles were identified among the many lesions isolated in a screen for small imaginal discs (Shearn and Garen 1974) and one in a screen for enhancers of *zen* (Hudson *et al*. 1998). Subsequently, additional *Med* alleles were isolated in F_2_ lethal screens. *Medea^13^* is a molecular null (Xu *et al*. 1998; Sutherland *et al*. 2003), and heterozygosity for *Medea* suppressed the wing phenotype produced by ectopic expression of a constitutively active Sax (Sax^Q263D^) but not activated Tkv^Q199D^ (Das *et al*. 1998). Embryos that are mutant for both the maternal and zygotic contributions of Medea lack amnioserosa, the dorsal-most cell fate, which cannot be rescued by the injection of either *dpp* mRNA or activated *Tkv* (*tkvA*) mRNA (Hudson *et al*. 1998). These data placed Medea downstream of activated BMP receptors, similar to Mad. The gene was cloned in parallel by 3 different labs (Das *et al*. 1998; Hudson *et al*. 1998; Wisotzkey *et al*. 1998) and found to be homologous to mammalian Smad4.

### Delineation of the signaling pathway

As components of the pathway were identified, the mechanics of transducing the ligand signal were simultaneously determined in both the BMP and Activin/TGF-β branches of the pathway. In short, ligand dimers are secreted into the extracellular space where they first bind to the ectodomain of their high-affinity S/T kinase transmembrane receptors. In the case of BMPs, they bind with high affinity to the type I receptor and then recruit the constitutively active type II receptors. The ectodomains of the BMP receptors are not thought to contact one another, and the final assembly consists of 2 type I and 2 type II receptors bound to the BMP ligand dimer (Bragdon *et al*. 2011). The formation of this ligand–receptor complex results in phosphorylation of the type I GS domain by the type II kinase. The now-activated BMP type I kinase phosphorylates the receptor-activated Smad (R-Smad), Mad in *Drosophila*, which complexes with co-Smad and Medea and regulates transcription with a variety of cofactors.

The different ligand and receptor combinations are thought to provide an array of different signaling outputs based on different affinities and stoichiometries of the individual components. The final heterohexameric complex consisting of a ligand dimer and 2 type I and 2 type II receptors, all of which are heteromeric, could result in a different level of Smad phosphorylation than a hexameric ligand–receptor complex composed of homodimer ligand and receptors. What controls the dimerization of the monomers to yield homodimer versus heterodimer ligands is still not well understood, nor is what determines how the ligand–receptor complex is assembled. While properties of the ligand and receptors themselves may drive different combinations that make a core signaling pathway, the large majority of ligands, type I receptors, type II receptors, and Smads, except for co-Smad and Smad4/Medea, tend to align with either the BMP or the Activin/TGF-β signaling branch. For example, in *Drosophila*, Tkv and Sax primarily mediate BMP signals to phosphorylate Mad, while Babo transduces Activin signals, phosphorylating Smox (dSmad2). However, both *Drosophila* type II receptors, Punt and Wit, clearly mediate signals from both branches.

Examples of such crossover between BMP and TGF-β/Activin signaling components are also apparent in vertebrate cells. As more studies are performed in vivo, it will become clear whether sharing of different components exhibits any common themes, such as being used in particular contexts. In *Drosophila*, Punt is thought to be the only functional type II receptor in the early embryo (Marqués *et al*. 2002), as well as in the wing imaginal disc where it signals with both Sax and Tkv (Nguyen *et al*. 1998; Bangi and Wharton 2006b). However, at the larval neuromuscular junction (NMJ), both Punt and Wit mediate signals, albeit *wit* whose expression and requirement appear to be limited to the presynapse/motor neuron with *punt* being required in the postsynaptic muscle membrane (Marqués *et al*. 2002; Fuentes-Medel *et al*. 2012). In addition, Punt and Wit assemble signaling complexes with both BMP type I receptors Tkv and Sax, as well as the Activin type I receptor Babo to mediate BMP (Gbb) and Activin (Act-B and Maverick) signals (Upadhyay *et al*. 2017). Beyond the differences between members of the ligand and receptor families, a number of common mechanisms have been revealed that regulate signaling output at different levels within the signal transduction pathway. Here, we highlight studies in *Drosophila*, which have informed a variety of ways in which BMP signaling is regulated.

#### Genetic modifier screens

As discussed above, genetic screens in *Drosophila* played a critical role in identifying the genes whose shared mutant phenotypes demonstrated their role in a common pathway. Subsequent screens for genetic modifiers, followed by epistasis studies, not only helped establish the core BMP signaling pathway but have also revealed a number of regulators. In one screen for zygotically acting, dominant enhancers of *dpp^hr4^*, novel lesions associated with haplolethality included 11 recessive alleles of *dpp*. Second site lesions included 2 alleles of *scw* and one allele of *tolloid* (*tld*). *tld* encodes a metalloprotease orthologous to mammalian BMP1, which was identified and named as one of the original factors purified from bone extract that first revealed the BMP ligands (Shimell *et al*. 1991; Raftery *et al*. 1995). *tld* had previously been identified in the embryonic patterning screens (Jurgens *et al*. 1984), and antimorphic alleles of *tld* had been shown to genetically interact with *dpp^hr^* alleles, affecting early DV patterning (Ferguson and Anderson 1992). Another screen for dominant enhancers of the tkv hypomorphic allele yielded 3 alleles of *gbb*, one allele of *tkv*, 2 of *punt*, 5 alleles of *Mad*, and one of *Medea* (Chen *et al*. 1998).

These early genetic modifier screens highlight some of the “go-to” developmental contexts used by researchers seeking to understand the mechanistic basis of BMP signaling and its regulation; these include DV patterning in the embryo, growth and patterning of the wing imaginal disc, and late wing vein patterning, specifically the formation of the posterior cross vein (PCV). Two other developmental contexts that have been used for a similar purpose: maintenance of the germ cell niche in both males and females and the growth and function of the larval NMJ have each provided an accessible tissue where BMP signaling is critical for its development and function. Together, the all-encompassing take-home message from the studies in these different systems is that *different molecular mechanisms regulate BMP signaling in different contexts*.

#### Receptor-mediated activation of Mad and transduction to the nucleus

With little insight as to *Mad*'s function from its sequence, the genetic demonstration that Mad acted downstream of the activated type I receptor Tkv^A^ allowed for the ordering of these pathway components and motivated intensive studies in both mammals and flies. It was shown that *Mad* is required for the constitutively active *TkvA* transgene to induce *dpp* target gene expression in multiple tissues (Wiersdorff *et al*. 1996; Newfeld *et al*. 1997; Hudson *et al*. 1998). Endogenous Mad protein was found to be predominantly cytoplasmic, even at sites of known Dpp activity (Newfeld *et al*. 1996), but it could be stimulated to accumulate in the nucleus by the addition of exogenous BMP2 or coexpression of activated type I receptor in cultured fly cells or by the transgenically elevated expression of Dpp in vivo (Maduzia and Padgett 1997; Newfeld *et al*. 1997). Studies of mammalian Mad homologs further delineated their role in signal transduction downstream of the activated BMP or TGF-β type I receptor [for a contemporaneous review, see Massagué (1998)].

#### Different types of Smads: R-Smad, Co-Smad, and iSmad

A growing understanding of the roles of mammalian Smads led to the division of the signal-transducing Smads into the Receptor-regulated Smad (R-Smad) family, which are phosphorylated by the activated type I receptor, and the common-mediator Smad, or co-Smad family, which bind to C-terminally phosphorylated R-Smads but are not themselves phosphorylated. Both R-Smads and co-Smads predominantly function to promote signal transduction by various TGF-β family members. Co-Smads can participate in either BMP or Activin signal transduction, through their association with the appropriate phosphorylated R-Smads (Table 1). Smads can form trimeric complexes with 2 phospho-R-Smads and one co-Smad; in vertebrates, hybrid complexes with both a BMP R-Smad and an Activin R-Smad have been detected (Inman and Hill 2002). Consistent with this, co-Smads have both MH1 and MH2 domains. A third group of Smads, the inhibitory-Smads (i-Smads), act as antagonists of signaling, retaining an MH2 domain, but with weak homology to MH1 domains (Hariharan and Pillai 2008). In *Drosophila*, Mad is the single R-Smad for the BMP signaling branch, dSmad2/Smox is the single R-Smad for the Activin branch (Henderson and Andrew 1998; Brummel *et al*. 1999), Medea is the single co-Smad, and Daughters against Dpp, or Dad, is the single i-Smad or inhibitory Smad (Tsuneizumi *et al*. 1997). In addition to *Drosophila* BMP signaling, Medea participates in the *Drosophila* Activin pathway (Brummel *et al*. 1999).

During embryonic DV patterning, nuclear Mad (phospho-Mad or pMad), as well as nuclear Medea, can be detected in the dorsal-most region of the presumptive amnioserosa (Eldar *et al*. 2002; Sutherland *et al*. 2003), a location where BMP signal activity is particularly strong (Shimmi, Umulis, *et al*. 2005; Wang and Ferguson 2005). Detection of endogenous nuclear Mad was achieved with the availability of antibodies against phosphorylated serine residues at the C-terminus (Dorfman and Shilo 2001). The use of anti-phosphoSmad1 antibodies remains the method of choice to detect cells that have received a BMP signal in situ and to assess the level of BMP activity between different *Drosophila* tissues, due to the great sensitivity of anti-pSmad1 compared with the detection of nuclear Medea (Sutherland *et al*. 2003).

Once in the nucleus, pMad/Medea complexes bind DNA. The conserved BMP-responsive DNA binding sites bound by Medea, Mad, and the trimeric Mad-Med were determined in *Drosophila* (Kim *et al*. 1996, 1997; Xu *et al*. 1998; Gao *et al*. 2005), while a distinct DNA binding site was revealed for the vertebrate Activin/Nodal/TGF-β Smad, Smad3 (Derynck *et al*. 1998; Kawabata and Miyazono 1999). A number of direct Mad targets have been determined in different contexts, highlighting the dual function of different Mad–Medea complexes. A precisely spaced combination of Mad and Medea binding sites mediate gene repression by a Mad–Medea–Schnurri complex (Dai *et al*. 2000; Pyrowolakis *et al*. 2004); initially, *schnurri* was implicated in BMP pathways through a shared requirement for embryonic D/V patterning (Arora *et al*. 1995; Dai *et al*. 2000). It is now clear that Mad can function as a transcriptional activator with or without Medea. In wing discs, Mad associates with Medea to activate *dad*, *omb*, and *sal* (Affolter *et al*. 2001), while Mad acts with Yki to activate *bantam* (Oh and Irvine 2011). In some cases, Mad and Medea activate the expression of a cofactor gene [*zerknüllt* (*zen*)] whose protein interacts with them to target downstream genes in a feed-forward mechanism (Xu *et al*. 2005). These are well-defined examples where the BMP response element has been dissected, and others are highlighted in later sections.

## Regulation of BMP signaling

### Regulation of BMP ligands

**Fig. 2.**
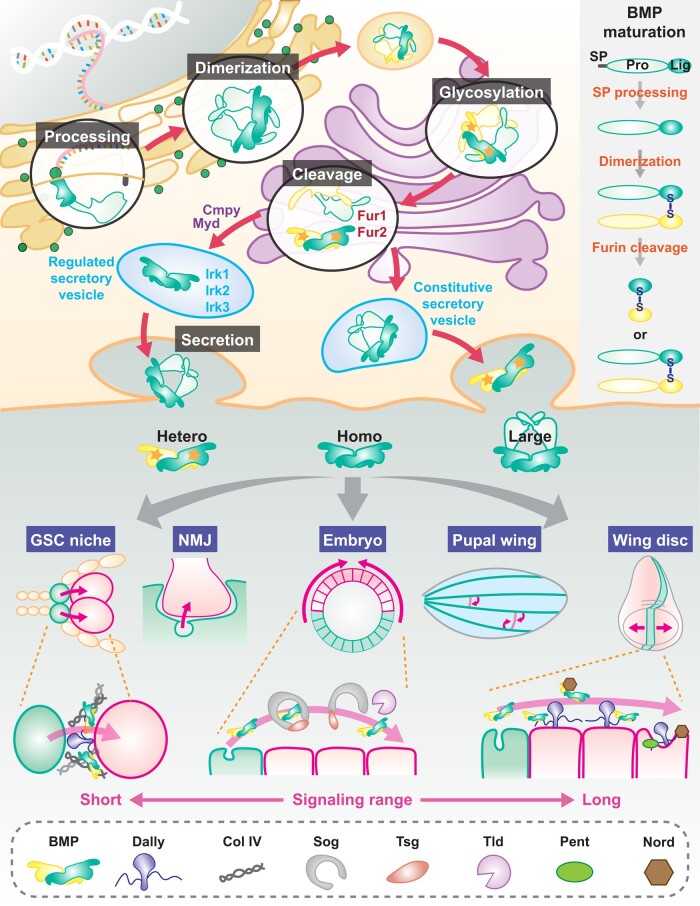
Regulation of BMP ligands. (Top) Posttranslational intracellular BMP regulation. Cell type–specific posttranslational modifications of inactive BMP proproteins occur in certain cellular organelles. After proper modifications, BMP producing cells secrete bioactive dimerized BMP ligands into the extracellular space. Several critical factors involved in this process are highlighted. The right column presents a simple diagram of BMP protein structures. (Bottom) Extracellular BMP regulation. Extracellular BMP interacting proteins control BMP ligand distribution in a context-dependent manner. GSC niche and NMJ exhibit a single-cell diameter BMP signaling range. On the other hand, BMP acts in intermediate and long distances during embryonic D/V patterning, PCV formation in the pupal wing, and longitudinal vein patterning in the developing wing disc.

The biological activity of BMP ligands is tightly controlled at 2 levels: (1) posttranslationally and secretion and (2) extracellularly by BMP interacting proteins (Fig. 2). *Drosophila* utilizes 3 BMP ligands, Dpp, Gbb, and Scw, to initiate signaling depending on the purpose and customizes ligand activity to regulate diverse biological processes ranging from developmental patterning to neurodevelopment and both germline and adult tissue homeostasis.

#### Posttranslational intracellular regulation

##### Proteolytic processing

Posttranslational regulation, such as proteolytic processing and glycosylation, controls both the quality and quantity of BMP ligands (Wozney *et al*. 1988; Akiyama *et al*. 2012; Wharton and Serpe 2013; Upadhyay *et al*. 2017). BMP family proteins are initially synthesized as inactive proproteins, which consist of a prodomain and a highly conserved ligand domain at the C-terminus. While the prodomains of different family members are less conserved, sequence comparisons show regions of conservation highlighting the evolutionary relatedness of specific ligand genes (Wisotzkey and Newfeld 2020). After translation, the proproteins dimerize via a disulfide bond between the ligand domains and undergo proteolytic processing to generate 110–140 amino acid bioactive BMP ligands comprised of the highly conserved C-terminal domain (Fig. 1). Dpp proprotein possesses 3 Furin proteolytic cleavage sites, an upstream FSII/S2 followed by FSIII/S1 and FSI sites (Kunnapuu *et al*. 2009; Sopory *et al*. 2010). It produces 2 mature ligands, Dpp26 (26 kDa) and Dpp23 (23 kDa), cleaved at FSIII/S1 and FSI sites, respectively (Kunnapuu *et al*. 2009; Akiyama *et al*. 2012). Biochemical and genetic analyses using various combinations of cleavage mutants demonstrate that the FSII/S2 site is essential for a long-range Dpp activity in the developing wing disc, but not for its short-range activity in the embryonic midgut (Kunnapuu *et al*. 2009; Sopory *et al*. 2010). Overexpression of a *dpp* mutant carrying an FSII/S2 mutation in the wing disc produces smaller amounts of mature ligands than that of wild-type *dpp^+^* (Kunnapuu *et al*. 2009; Sopory *et al*. 2010), indicating that this upstream cleavage site is required for normal ligand production in the wing disc by either affecting subsequent FSIII/S1 or FSI cleavage or influencing ligand stability. As expected, this mutant neither forms a long-range extracellular gradient nor properly activates the pathway and fails to rescue the *dpp* hypomorphic (*disc* alleles) wing phenotype. In contrast, in the embryonic midgut where Dpp has a short-range activity, exogenous expression of wild-type *dpp^+^*, this same mutant produces comparable amounts of mature ligands and exhibits a similar BMP signaling capability (Sopory *et al*. 2010). These results suggest that tissue-specific differential Dpp proteolytic processing contributes to the establishment of distinct ranges of Dpp action controlled by ligand production.

The 2 other BMPs, Scw and Gbb, have quite different proteolytic processing properties (Akiyama *et al*. 2012; Fritsch *et al*. 2012; Künnapuu *et al*. 2014; Anderson and Wharton 2017). In addition to the 2 conventional cleavage sites adjacent to the ligand domain, they also have processing site(s) within the prodomain. Scw proprotein contains 4 proteolytic processing sites: 2 in the prodomain, Pro2/FSII and Pro, and 2 just N-terminal to the ligand domain, Main/FSI and Shadow sites (Fritsch *et al*. 2012; Künnapuu *et al*. 2014). Among them, Pro and Main/FSI are essential for *scw* function. A mutation in only the Main/FSI site generates a nonfunctional Scw ligand possessing a large portion of its prodomain, while a Pro mutant generates a prodomian–ligand complex, which inhibits proper Scw signaling activity. Expectedly, both mutants are unable to rescue embryonic lethality caused by *scw* null mutations (Fritsch *et al*. 2012). It is notable that the antimorphic *scw^E1^* allele, which enhances the *dpp* hypomorphic embryonic lethal phenotype, carries a point mutation at the Pro processing site (Raftery *et al*. 1995; Künnapuu *et al*. 2014). Scw^E1^ protein preferentially forms a heterodimer with Dpp and interferes with its signaling activity by forming an unprocessed Scw prodomain-associated heterodimer complex (Künnapuu *et al*. 2014). Unlike Scw, cleavage of proGbb at either its prodomain site, NS/Pro, or its conventional sites, S1/Main and S0/Shadow, adjacent to the ligand domain can rescue *gbb* mutant lethality, with each producing 2 totally different sizes of bioactive Gbb ligands, Gbb15 and Gbb38 (Fig. 1; Akiyama *et al*. 2012; Fritsch *et al*. 2012; Anderson and Wharton 2017). In addition to the small Gbb ligand, Gbb15 (15 kDa), generated by conventional site proteolytic processing, cleavage of Gbb proprotein at only the NS/Pro site produces a larger Gbb38 ligand (38 kDa) with distinct signaling properties (Akiyama *et al*. 2012; Fritsch *et al*. 2012; Anderson and Wharton 2017). Just as in Scw, NS/Pro cleavage is important to liberate Gbb15 from the prodomain for promoting its signaling activity (Anderson and Wharton 2017). Therefore, NS/Pro cleavage has a dual function that regulates Gbb15 activity and produces Gbb38. In vivo functional assays show that NS/Pro cleavage is required for wing vein patterning and pupal ecdysis (Anderson and Wharton 2017). Since Gbb proteolytic processing is regulated in a tissue-dependent manner (Akiyama *et al*. 2012), differential cleavage may be responsible for context-specific signaling outputs. Studies in S2 cells indicate that Dpp can likely form heterodimers with both Gbb15 and Gbb38 (Anderson and Wharton 2017). Altogether, despite their similar protein structures, Scw and Gbb exhibit different requirements of alternative proteolytic processing for regulating BMP signaling.

##### Other posttranslational modifications

Glycosylation plays a critical role in modulating BMP signaling activity (Fig. 2). Recent work has demonstrated that O-glycosylation influences proteolytic processing at specific sites within the proprotein (Anderson and Wharton 2017). In 3rd instar larvae, Gbb38 is the most abundant ligand. In close proximity to conventional S1/Main and S0/Shadow cleavage sites, O-glycosylation blocks proteolytic processing at these sites resulting in cleavage of the Gbb proprotein at only the NS/Pro site, producing the large form of Gbb, Gbb38.

In addition to O-glycosylation, N-glycosylation of the Scw ligand has been shown to influence Scw dimer formation, secretion, and signaling activity (Tauscher *et al*. 2016). Scw contains 2 *N*-glycosylation sites: N342 is highly conserved between BMP2/4Dpp and BMP5/6/7/8/Gbb, whereas another site, N304, is specific to Scw. Although both sites are crucial for Scw function, blocking *N*-glycosylation at the conserved site exhibits a stronger reduction in BMP signaling activity both in vitro and in vivo, compared with a Scw-specific *N*-glycosylation site mutant, *scw^N304Q^*, and a minimal rescue of *scw* lethal phenotype. Interestingly, despite its weaker impact on Scw function, a loss of *N*-glycosylation at this unique site results in the preferential generation of Dpp/Scw heterodimers in vitro, while a mutation at the conserved site, *scw^N342Q^*, produces comparable amounts of heterodimers as *wild-type* Scw. This finding is of particular interest because little is known of what molecular mechanisms drive whether BMP monomers expressed within the same cell form homodimers vs heterodimers, despite the different signaling outputs and the importance of one form over the other in many developmental processes. Furthermore, Scw ligands are not efficiently secreted when both cleavage sites are mutated and presumably as would be the case if cleavage was blocked at both sites.

GlcNAcylation, mediated by *mummy* (*mmy*), encoding the *Drosophila* UDP-GlcNAc pyrophosphorylase, has been shown to restrict the range of Dpp-induced signaling in the epidermal leading edge during dorsal closure of the embryo (Humphreys *et al*. 2013). In this case, the modification of the Dpp protein is thought to spatially limit its action.

##### Secretion

Secretion of BMP ligands into the extracellular space is critical for their interaction with receptor ectodomains. While the role of extracellular antagonists as regulators of ligand availability for receptor binding has been well-recognized and studied (see below), at present we have a limited understanding of how intracellular trafficking and secretion of BMPs are regulated. However, several *Drosophila* studies have given us some important insights. A report showing that *lethal(2) giant larvae* (*lgl*), the ortholog of yeast sro7/77, a factor important in polarized exocytosis, is required in Dpp-producing cells; upstream of the Tkv receptor for full *dpp* function is suggestive of a role for *lgl* in Dpp secretion (Arquier *et al*. 2001). More recently, inwardly rectifying potassium (Irk) channels have been shown to influence Dpp secretion (Dahal *et al*. 2017). Both mutations in *irk2* and overexpression of dominant-negative forms of Irk2 lead to a severe loss of BMP signaling activity in the developing wing disc, resulting in wing patterning defects (Dahal *et al*. 2012, 2017). Irk2 depolarizes Dpp-producing cells, increasing intracellular Ca^2+^ concentration and stimulating Dpp secretion (Dahal *et al*. 2017).

Similarly, it has been shown that when overexpressed in motor neurons, the secretion of coexpressed Gbb depends on Ca^2+^ influx (James *et al*. 2014). A BMP binding protein, Crimpy, can direct Gbb expressed in motor neurons to dense core vesicles for a neuronal activity-dependent release from the presynaptic cells. Crimpy can form an extracellular complex with Gbb, thereby distinguishing 2 pools of Gbb at the NMJ: presynaptic Gbb/Crimpy that appears to act in baseline neurotransmitter release and postsynaptic Gbb produced by the muscle which controls NMJ growth.

Two other factors affecting Gbb release are Cdc-42–interacting protein 4 (dCIP4; Nahm *et al*. 2010) and the Golgi luminal protein Mayday, a *Drosophila* Cad45 homolog (Sidisky *et al*. 2021). Gbb release from muscle is inhibited by dCIP4, but its secretion from dorsal longitudinal muscles is promoted by Mayday. *mayday* mutants cause the accumulation of Gbb in postsynaptic muscles, with a decrease in BMP signaling activity in the presynaptic motor neurons, resulting in a progressive loss of proper synaptic structures and flight ability (Sidisky *et al*. 2021).

Together, these reports indicate that changes in ligand processing and intracellular trafficking can alone influence signaling output. Thus, a more thorough understanding of the mechanisms at play in regulating the in vivo production of bioactive BMP ligands across metazoans is warranted in order to appreciate the full potential of these versatile signaling molecules.

#### Extracellular regulation by BMP interacting proteins

After secretion, biological activity of BMP ligands is further controlled in a tissue-dependent manner by secreted BMP inhibitors, metalloproteases, and extracellular matrix proteins such as Collagen IV and heparan sulfate proteoglycans (HSPGs; Fig. 2; O’Connor *et al*. 2006; Affolter and Basler 2007; Yan and Lin 2009; Harris *et al*. 2011; Raftery and Umulis 2012; Ramel and Hill 2012; Shimmi and Newfeld 2013; Wharton and Serpe 2013; Hamaratoglu *et al*. 2014; Restrepo *et al*. 2014; Akiyama and Gibson 2015; Bier and De Robertis 2015; Nakato and Li 2016; Kamimura and Maeda 2017; Upadhyay *et al*. 2017). Such extracellular regulation endows a context-specific BMP signaling activity during development and adult homeostasis. For instance, 2 distinct modes of BMP ligand behaviors, i.e. their “accumulation” and “dispersal”, which are controlled by extracellular BMP binding proteins, are critical for the generation of BMP morphogen activity gradients. Accumulation of ligands through a facilitated transport is essential for the embryonic D/V patterning and posterior crossvein (PCV) formation (Raftery and Sutherland 2003; O’Connor *et al*. 2006; Ramel and Hill 2012; Shimmi and Newfeld 2013; Wharton and Serpe 2013; Akiyama and Gibson 2015; Bier and De Robertis 2015; Upadhyay *et al*. 2017). However, in the developing wing disc, ligand dispersal creates the morphogen gradient that patterns the adult wing (Affolter and Basler 2007; Ramel and Hill 2012; Wharton and Serpe 2013; Hamaratoglu *et al*. 2014; Restrepo *et al*. 2014; Akiyama and Gibson 2015; Upadhyay *et al*. 2017).

##### Spatiotemporal control of ligand accumulation

###### Morphogen gradient formation in the early embryo

During D/V patterning in early embryos, secreted BMP antagonists, Short gastrulation (Sog) and Twisted gastrulation (Tsg), and BMP-1 metalloprotease Tolloid (Tld) play critical roles in facilitated BMP transport (Raftery and Sutherland 2003; O’Connor *et al*. 2006; Ramel and Hill 2012; Shimmi and Newfeld 2013; Wharton and Serpe 2013; Akiyama and Gibson 2015; Bier and De Robertis 2015; Upadhyay *et al*. 2017). Dorsally produced Dpp and uniformly expressed Scw appear to form 3 distinct BMP ligands, Dpp and Scw homodimers, and Dpp/Scw heterodimer, and their differential activities are thought to be required for the embryonic D/V patterning (Shimmi, Ralston, *et al*. 2005). Among them, Dpp/Scw heterodimers are considered the primary transport ligands responsible for the establishment of a peak BMP activity at the dorsal midline by the following experimental evidence: (1) both *dpp* and *scw* null mutants die as ventralized embryos (Arora and Nüsslein-Volhard 1992; Ferguson and Anderson 1992; Wharton *et al*. 1993; Arora *et al*. 1994), (2) Dpp homodimers fail to accumulate in the dorsal midline in *scw* mutants (Shimmi, Umulis, *et al*. 2005; Wang and Ferguson 2005), and (3) Dpp/Scw heterodimers have a higher affinity for Sog and Tsg than the homodimers, stimulate Sog processing by Tld, and possess significantly stronger signaling activity compared with the homodimers (Shimmi, Umulis, *et al*. 2005). Further, consistent with a critical requirement of heterodimers, dorsal-specific expression of *scw* induced by a *tld* promoter is able to rescue the *scw* mutant phenotype (Arora *et al*. 1994).

In the current model, a Dpp/Scw heterodimer and its antagonist Sog independently bind the scaffold protein Collagen IV in the dorsolateral region (Wang *et al*. 2008; Sawala *et al*. 2012). These interactions are mediated by an N-terminally located basic amino acid motif in the Dpp ligand and the Sog cysteine-rich (CR) domains (Sawala *et al*. 2012). It is worth noting that Collagen IV only interacts with Dpp, but not Scw (or Gbb) due to a lack of the motif. On the scaffolding protein, Dpp/Scw forms a complex with Sog by remodeling their protein interactions. Then, additional Tsg interaction releases the Dpp/Scw–Sog–Tsg shuttling complex from Collagen IV (Sawala *et al*. 2012). Since Dpp/Scw within the complex cannot interact with BMP receptors, Sog–Tsg facilitates the dorsal transport of the BMP heterodimer. Tsg interaction also enhances Sog cleavage by Tld metalloprotease to liberate Dpp/Scw from the inhibitory complex for either reforming the shuttling complex or for interacting with signaling receptors (Shimmi and O’Connor 2003).

A recent study shows that Collagen IV also interacts with Tld and enhances its protease activity (Winstanley *et al*. 2015). Further, Sog diffuses dorsally from the ventral side of the embryo where it is produced, and the concentration is gradually diminished toward the dorsal most cells by Tld-dependent degradation and Dynamin-dependent retrieval (Srinivasan *et al*. 2002). Another dorsally produced BMP-1 metalloprotease Tolloid-related (Tlr) is also involved in generating the Sog gradient, although *tlr* mutants are not embryonic lethal (Nguyen *et al*. 1994; Finelli *et al*. 1995; Srinivasan *et al*. 2002; Meyer and Aberle 2006). Thus, in the dorsolateral region, free Dpp/Scw heterodimers likely reform the shuttling complex due to a high Sog concentration (Srinivasan *et al*. 2002). The sequential reactions of the shuttling complex formation, Sog cleavage by Tld and BMP liberation, facilitate Dpp/Scw accumulation at the dorsal midline. Liberated Dpp/Scw ligands elicit a peak of high BMP signaling activity that specifies the dorsal-most cells as amnioserosa, while in the dorsolateral cells, lower levels of BMP signaling activity, presumably triggered by homodimers, lead to the specification of dorsal ectoderm (Ferguson and Anderson 1992; Wharton *et al*. 1993; Dorfman and Shilo 2001; Ross *et al*. 2001; Sutherland *et al*. 2003; Mizutani *et al*. 2005; Shimmi, Ralston, *et al*. 2005; Wang and Ferguson 2005; Wharton and Serpe 2013; Upadhyay *et al*. 2017).

Last, it has been reported that another extracellular protein, Crossveinless-2 (Cv-2), antagonizes BMP signaling in the early embryo by interacting with BMP via its CR domains and a C-terminal von Willebrand factor D domain (Serpe *et al*. 2008; Gavin-Smyth *et al*. 2013). *cv-2* forms a genetic circuit with at least 2 other genes, *zen* and *eiger*, and contributes to the robustness/canalization of BMP signaling during D/V pattern formation (Gavin-Smyth *et al*. 2013; Gavin-Smyth and Ferguson 2014). While the Cv-2 function is essential for PCV formation (described below), Cv-2 produced by either maternally or zygotically is not absolutely required for embryogenesis, since *cv-2* null mutants can be maintained as homozygotes in the laboratory condition (Conley *et al*. 2000; Serpe *et al*. 2008). While not essential in the embryo, the buffering function of Cv-2 in DV patterning may be exerted under particular genetic and environmental conditions.

###### Ligand transport during PCV development in the pupal wing

In the formation of the PCV, Dpp and Gbb play essential roles. *dpp* expression is initially detected only in the longitudinal vein regions when BMP transport is actively taking place, but at a later stage, it is also found in the PCV region (Yu *et al*. 1996; de Celis 1997; Ralston and Blair 2005). In contrast, Gbb is produced in a largely uniform manner (Conley *et al*. 2000). Slightly different shuttling components, Sog, Tsg2/Cv, and Tlr, are employed during this process (O’Connor *et al*. 2006; Ramel and Hill 2012; Wharton and Serpe 2013; Upadhyay *et al*. 2017). However, just like in the early embryo, the heterodimer, in this case, Dpp/Gbb, is the preferred interacting partner of the Sog-Tsg2/Cv complex, and it efficiently migrates into the presumptive PCV region from the primordial longitudinal vein cells (Shimmi, Umulis, *et al*. 2005; Matsuda and Shimmi 2012). Supporting this idea, (1) *dpp* (*shortvein* alleles) and *gbb* hypomorphic mutants cannot form the PCV, (2) null mutant clones that largely occupy the longitudinal veins adjacent to the PCV lead to a crossvein defect (de Celis 1997; Haerry *et al*. 1998; Khalsa *et al*. 1998; Ray and Wharton 2001), (3) longitudinal vein-specific induction of *gbb* can rescue its mutant phenotype (Matsuda and Shimmi 2012), and (4) Dpp homodimers are not delivered to the PCV region in a *gbb* mutant background (Matsuda and Shimmi 2012). The directional transport of the active BMP ligand is facilitated by a nonuniform Sog distribution, higher in intervein cells and lower in expression of the developing PCV region, generated via a BMP signaling-independent mechanism (Ralston and Blair 2005; Matsuda and Shimmi 2012). Indeed, uniform or posterior-specific Sog overexpression leads to a loss of BMP signaling activity in the presumptive PCV cells and causes a complete lack of PCV in adult wings (Yu *et al*. 2004; Ralston and Blair 2005; Serpe *et al*. 2008).

Overexpression of an uncleavable form of Sog exhibits stronger effects than *wild-type* Sog (Peluso *et al*. 2011), indicating the importance of Sog cleavage for proper PCV formation. Additionally, a recent study identifies *N*-glycosylation sites in Sog and shows that a loss of *N*-glycosylation enhances its antagonistic activity in both early embryos and pupal wings (Negreiros *et al*. 2018). Although the PCV formation utilizes a similar facilitated transport mechanism, there are several differences. Unlike the Dpp/Scw heterodimer, in this context, the Dpp/Gbb heterodimer has a comparable signaling capability than the homodimers (Shimmi, Umulis, *et al*. 2005). Tlr possesses a slower kinetic of Sog cleavage than Tld (Serpe *et al*. 2005). Along with this observation, they are unable to substitute for each other in rescue experiments (Nguyen *et al*. 1994; Serpe *et al*. 2005), suggesting a tissue-specific functional adaptation of BMP-1 metalloproteases.

###### Additional extracellular modulators regulate ligand availability

As compared with the embryo, Collagen IV does not seem to be actively involved in this process (Matsuda *et al*. 2013). Instead, other extracellular proteins, Cv-2, Cv-C, Cv-D, Larval Translucida (Ltl), and HSPGs, are required for proper PCV formation (Diaz-Benjumea and Garcia-Bellido 1990; Conley *et al*. 2000; Ralston and Blair 2005; Serpe *et al*. 2008; Szuperak *et al*. 2011; Chen *et al*. 2012; Karim *et al*. 2012; Matsuda and Shimmi 2012). Interestingly, most of these extracellular proteins interact and function with HSPGs. HSPGs consist of a protein core and highly modified HS chains and are categorized into 3 major groups based on the core protein structures: secreted perlecan, transmembrane syndecan, and membrane-tethered glypican (Yan and Lin 2009; Nakato and Li 2016; Kamimura and Maeda 2017). HSPGs interact with many growth factors including BMPs through both a protein core and HS chains (Kirkpatrick *et al*. 2006; Akiyama *et al*. 2008; Kanai *et al*. 2018). Secreted Cv-2 proteins localize on the cell surface mainly by interacting with HSPGs, such as the glypicans, Division abnormally delayed (Dally), and Dally-like (Dlp), through HS chains (Serpe *et al*. 2008). Membrane-localized Cv-2 acts as both a short-range agonist and antagonist depending on the concentration of Cv-2 and BMP ligands. In addition, the type of BMP ligand, Dpp or Gbb homodimer, influences this biphasic activity of Cv-2 in vitro. However, how this difference impacts Cv-2 activity in vivo is unclear, because the Dpp/Gbb heterodimer, but not the homodimers, appears to be the primary ligand form during PCV development. On the cell surface, Cv-2 interacts with BMP ligands released from the Sog–Tsg2/Cv complex to either build the inhibitory complex or transiently form the exchanging complex with BMP type I receptor, such as Tkv, to transfer BMP ligands for signaling (Serpe *et al*. 2008). Since Cv-2 expression itself is regulated by BMP signaling, both BMP-dependent Cv-2 expression and its protein dynamics modulate the biphasic Cv-2 activity to ensure proper PCV development.

Another secreted BMP feedback regulator Ltl, which is expressed in the longitudinal and crossvein regions, physically binds to Dlp and genetically interacts with *cv-2* (Szuperak *et al*. 2011). Ltl acts as a BMP antagonist when overexpressed and leads to a lack of the PCV. Interestingly, although both the loss of *cv-2* and the overexpression of *ltl* cause the same PCV loss phenotype, leaky exogenous *ltl* expression (weak *ltl* overexpression from a UAS transgenic line) rescues the *cv-2* mutant phenotype in a dose-dependent manner. This rescue experiment suggests that *ltl* and *cv-2* have partially redundant functions in PCV development. However, the molecular basis underlying their cooperation is unclear.

*cv-d* encodes a vitellogenin-like lipoprotein that, unlike other crossvein-less group proteins, functions remotely to control the PCV formation (Chen *et al*. 2012). Cv-D proteins in the developing pupal wing are largely supplied by the fat bodies via hemolymph and are proposed to act as another BMP transporter by interacting with both BMP and HSPGs. Mechanistically, how this transporter and the Sog-Tsg2/Cv shuttling complex work together to achieve normal PCV development remains elusive.

Integrins are also involved in this process (Araujo *et al*. 2003; Matsuda and Shimmi 2012). They genetically interact with *sog* and modulate Sog activity by affecting its distribution in the developing pupal wing (Araujo *et al*. 2003). Recent work has reported that BMP signaling induces the expression of *cv-c*, which encodes Rho GTPase-activating protein, in the developing PCV region (Matsuda *et al*. 2013). Cv-C regulates tissue morphogenesis (lumen formation) at the PCV region by inactivating Rho family GTPases, such as Rho1 and Cdc42, and by downregulating b-Integrin levels at the luminal side. Tissue morphogenesis mediated by Cv-C promotes BMP transport. Thus, Cv-C acts as a key regulator to couple tissue morphogenesis and the directional BMP transport through BMP feed-forward loop regulation. As described, to accomplish a spatiotemporally controlled accumulation of BMP ligands, the early embryo and pupal wing leverage similar, but quite different, molecular mechanisms by using both common and distinct extracellular BMP modulators. These machineries may have evolved successfully to control the response to BMP distribution to different developmental conditions, which involve different time constraints for the establishment of BMP activity gradients, 30 min in the early embryo (Ross *et al*. 2001; Wang and Ferguson 2005; Shimmi, Umulis, *et al*. 2005) versus several hours in the developing pupal wing (Conley *et al*. 2000; Serpe *et al*. 2005; Matsuda *et al*. 2013; Gui *et al*. 2016) or distinct extracellular environments.

##### BMP dispersal controlled by extracellular proteins

###### Long-range morphogen activity gradient in developing wing

In the developing wing disc, 2 BMP ligands, Dpp and Gbb, generate a gradient of BMP activity centered at the anterior-posterior (A/P) compartment boundary and visualized by a gradation in nuclear pMad. The BMP activity gradient patterns the wing disc through the activation and repression of target genes, *spalt* (*sal*), *optomotor blind* (*omb*), and *brinker* (*brk*), establishing discrete spatial domains where vein and intervein primordial cell fates are specified (Affolter and Basler 2007; Hamaratoglu *et al*. 2014; Restrepo *et al*. 2014; Akiyama and Gibson 2015; Upadhyay *et al*. 2017). *dpp* is expressed and produced by a stripe of anterior cells abutting the A/P compartment boundary (Posakony *et al*. 1990; Raftery *et al*. 1991), while *gbb* is expressed more broadly with lower levels in the central stripe where *dpp* is expressed (Khalsa *et al*. 1998). The requirement for both *dpp* and *gbb* in generating the BMP activity gradient is clear. When *dpp* expression is eliminated from its expressing cells, the activity gradient is lost and the target gene expression is severely disrupted (Akiyama and Gibson 2015; Barrio and Milán 2017; Bosch *et al*. 2017; Matsuda and Affolter 2017). Detailed mosaic mutant analyses show that *gbb* null clones lead to wing defects, with anterior clones overlapping the *dpp* stripe region, producing more severe wing defects (Khalsa *et al*. 1998; Ray and Wharton 2001; Bangi and Wharton 2006a).

Wing discs with such clones, devoid of anterior *gbb* function, fail to form a proper BMP activity gradient (Bangi and Wharton 2006a). These results indicate that in the absence of Gbb, Dpp alone is unable to form a long-range BMP morphogen gradient, providing functional evidence that Dpp/Gbb ligand heterodimers may play a critical role in gradient formation. It has been technically extremely challenging to differentiate between homodimers and heterodimers in vivo. This has made it difficult in all systems to attribute specific functions to a particular ligand form (homodimer vs heterodimer). With regard to wing patterning, a recent study leveraged innovative genetic manipulations to address the contributions of specific ligand types to the generation of the BMP morphogen gradient (Bauer *et al*. 2023). First, small epitope tags were introduced into the *dpp* and *gbb* loci via CRISPR/Cas9-mediated gene editing, allowing for visualization of endogenous Dpp or Gbb expression. Despite the nearly uniform expression of *gbb* across the wing pouch and the restricted expression of *dpp* to the narrow stripe of cells along the A/P compartment boundary, each tagged extracellular BMP ligand showed a similar graded distribution centered at the A/P boundary. Second, a synthetic morphotrap that captures either Dpp or Gbb in the extracellular space allowed for the demonstration of Dpp/Gbb heterodimers in vivo. Last, experiments that knockdown *dpp* revealed that the secretion of Gbb depends on Dpp indicating that the Gbb/Dpp heterodimer is the primary form of secreted ligand emanating from the A/P boundary. These data provide an important in vivo molecular demonstration of conclusions drawn using conventional genetic approaches (Khalsa *et al*. 1998; Ray and Wharton 2001; Bangi and Wharton 2006a). Together, these studies provide strong in vivo evidence for BMP heterodimer function. They also highlight that it is critical to know the subcellular distribution and active state of endogenous BMP ligands in specific contexts before a complete understanding of the action of these potent signaling molecules can be attained.

Although the molecular mechanisms underlying BMP morphogen gradient formation are still debated, it is clear that HSPGs, such as Dally, play essential roles in creating proper gradient (Affolter and Basler 2007; Hamaratoglu *et al*. 2014; Restrepo *et al*. 2014; Akiyama and Gibson 2015; Upadhyay *et al*. 2017). The *dally* locus was initially identified in a genetic screen designed to discover genes required for cell cycle regulation in the developing central nervous system (CNS) using homozygous viable enhancer trap lines, but *dally* mutants not only affect the cell division pattern in the larval CNS but also exhibit pleiotropic adult phenotypes, including a small eye and wing venation defects, by affecting multiple growth factor signaling pathways including BMP (Nakato *et al*. 1995). Further studies demonstrate that *dally* mutation affects the expression of BMP target genes in the developing eye and wing discs (Nakato *et al*. 1995; Jackson *et al*. 1997; Fujise *et al*. 2001).

###### Extracellular modulation of long-range BMP activity

In the wing disc, Dally play a critical role in the establishment of the BMP gradient. In a *wild-type* background, when GFP-Dpp proteins are exogenously overexpressed in the A/P boundary stripe cells, GFP-Dpp migrates laterally to form a concentration gradient. In contrast, little GFP-Dpp is detectable outside of the overexpressing cells in *dally* mutant wing discs, suggesting that Dally is essential for Dpp dispersal. As expected, a *dally* mutant disc has a narrower BMP activity gradient, leading to the same L5 wing vein defect that is observed in *gbb* mutants (Nakato *et al*. 1995; Fujise *et al*. 2001, 2003; Akiyama *et al*. 2008; Dejima *et al*. 2011). Clonal analyses of null alleles of *dally* and another glypican, *dlp* (*dally-like protein*), reveal the cell autonomous requirement of these proteins in the establishment of a BMP gradient (Belenkaya *et al*. 2004). While *dally* mutant clones show a significant reduction in BMP activity, as well as an L5 wing vein defect, *dlp* mutant clones show no obvious adult wing venation phenotype (Han *et al*. 2004). However, *dally dlp* double mutant clones exhibit a more severe effect on BMP signaling than *dally* single mutant cells, suggesting that these glypicans have partially redundant functions in wing patterning (Belenkaya *et al*. 2004). This study also revealed a local nonautonomous effect, in that BMP signaling activity is maintained in the first row of double mutant cells adjacent to the BMP source (Belenkaya *et al*. 2004). This ability to signal is thought to be due to a trans activity of glypicans, by which glypicans on the *wild-type* cell surface act in trans as coreceptors to support BMP signaling in the glypican-deficient cells (Hayashi *et al*. 2009; Dejima *et al*. 2011).

Two distinct genetic approaches highlight the critical requirement for heparan sulfate (HS) modification for proper BMP morphogen gradient formation. First, extracellular Dpp movement and BMP signaling activity are severely impaired in the clones of cells lacking the ability to produce HS chains (Bornemann *et al*. 2004; Han *et al*. 2004; Takei *et al*. 2004). In addition, manipulating the composition of HS chains by either generating HS-modifying enzyme mutant cells or overexpressing the enzymes significantly affects BMP signaling, suggesting that not only HS chains but also particular HS modifications are required for the gradient formation (Kamimura *et al*. 2011; Dejima *et al*. 2013). Second, as an alternative strategy, a mutant form of Dpp, which lacks N-terminal 7 basic amino acid residues essential for heparin and Dally binding, is employed (Akiyama *et al*. 2008). The Dpp mutant protein has a shorter protein half-life both in vitro and in vivo than *wild-type* Dpp, and it fails to form an extracellular gradient when overexpressed in the *dpp-*expressing cells. Moreover, a genetic interaction study between *dally* and *tkv* reveals their opposing effects on gradient formation: the gradient is shrunk in *dally* mutants, while it is extended in *tkv* mutants. When they are combined, the gradient is somewhat restored (Akiyama *et al*. 2008). Since Tkv is proposed to regulate extracellular Dpp levels by receptor-mediated endocytosis (Entchev *et al*. 2000; Belenkaya *et al*. 2004), Dally may promote the long-range BMP morphogen gradient formation by antagonizing receptor-mediated degradation (Akiyama *et al*. 2008). Supporting this idea that Dally stabilizes Dpp on the cell surface, ectopic overexpression of Dally, but not Dlp, enhances BMP signaling (Fujise *et al*. 2003; Takeo *et al*. 2005; Dejima *et al*. 2011). It is also worth to note that, although a mutant form of Dally lacking HS chains retains some ability to interact with Dpp, overexpression of this HS chain-deficient Dally is unable to promote BMP signaling (Kirkpatrick *et al*. 2006), again highlighting the requirement of HS chains for BMP signaling.

###### Role of transcriptional feedback in activity gradient

Transcriptional feedback regulation is critical in establishing and maintaining proper BMP activity gradient in the developing wing disc. Previous microarray and genome-wide in silico screening studies identify 2 secreted BMP feedback regulators, Pentagone (Pent) and Larval translucida (Ltl), essential for proper gradient formation (Vuilleumier *et al*. 2010; Szuperak *et al*. 2011). *ltl* expression is induced by BMP signaling and exhibits an antagonistic activity (Szuperak *et al*. 2011). Conversely, BMP signaling represses *pent* expression in the central region similar to *brk*. Laterally produced Pent promotes the formation of BMP gradient (Vuilleumier *et al*. 2010). Despite their functions, intriguingly, neither Ltl nor Pent binds Dpp (Vuilleumier *et al*. 2010; Szuperak *et al*. 2011). Recent work demonstrates that Pent controls BMP signaling by regulating glypican availability on the cell surface via Dynamin-dependent and Rab5-dependent internalization (Norman *et al*. 2016). Since Ltl physically interacts with HSPGs, it may exert its antagonistic activity through HSPGs via a yet unknown mechanism. Recent studies identified additional extracellular BMP feedback regulator Nord (Akiyama *et al*. 2022; Yang *et al*. 2022). *nord* expression at the A/P compartment boundary is positively regulated by BMP and Hh signaling. Nord fine-tunes BMP signaling via 2 distinct molecular actions. First, Nord physically interacts with BMPs and shows a higher affinity for Dpp/Gbb heterodimer; Nord regulates BMP signaling in a biphasic manner, promoting the pathway at low levels but inhibiting it at high concentrations (Yang *et al*. 2022). Second, like Pent, Nord physically binds to Dally and destabilizes it via endocytosis-mediated degradation, thus negatively regulating BMP signaling output (Akiyama *et al*. 2022). Given that Dally expression is also controlled by BMP signaling (Fujise *et al*. 2003), BMP feedback regulation may assist robust BMP morphogen gradient formation via its ability to balance BMP agonistic and antagonistic proteins in the extracellular space.

Last, several studies have found other extracellular proteins that regulate BMP signaling in the developing wing disc. A recent study investigates the roles of basement membrane proteins, Collagen IV and secreted HSPG Trol, in BMP signaling during wing development (Ma, Cao, *et al*. 2017). While Trol does not seem to affect BMP signaling based on the finding that the Trol RNAi animals can develop normal adult wings, disrupting Collagen IV function by RNAi leads to a dramatic reduction of Dpp protein levels in the wing disc and causes a loss of BMP signaling activity. This result suggests that Collagen IV proteins function as a barrier and block Dpp diffusion in wing disc epithelia to maintain proper BMP signaling activity. In addition, overexpression studies suggest potential roles for Follistatin (Fs) and the transmembrane HSPG, Syndecan, in BMP signaling (Bickel *et al*. 2008; Pentek *et al*. 2009; Yamamoto-Hino *et al*. 2015). Fs is prominently expressed in the wing disc, and its overexpression strongly downregulates BMP target gene expression in the wing disc, indicating its potential to antagonize BMP activity (Bickel *et al*. 2008; Pentek *et al*. 2009). Overexpressing Syndecan in the developing wing disc leads to a thick vein phenotype reminiscent of BMP signaling defects (Yamamoto-Hino *et al*. 2015). An examination of the endogenous functions of these proteins on BMP signaling will clarify their putative roles as regulators of signaling output.

#### Developmental regulation by ligand

##### Short-range BMP signaling for stem cell maintenance

Many morphogens, including BMP, also function as factors in the stem cell niche regulating stem cell behaviors. In the ovary, 2 BMP ligands, Dpp and Gbb, act as short-range signaling molecules to regulate germline stem cell (GSC) self-renewal (Xie and Spradling 2000; Song *et al*. 2004; Chen *et al*. 2011; Upadhyay *et al*. 2017). Both ligands are expressed in the niche cells (cap and/or GSC contacting escort cells) located at the anterior tip of the germarium. Dpp is expressed in both cell types with a stronger expression in cap cells, while Gbb is expressed at least in escort cells (Xie and Spradling 2000; Song *et al*. 2004; Rojas-Rios *et al*. 2012; Ma *et al*. 2014; Liu *et al*. 2015). BMP ligands produced by the niche cells activate only GSCs and maintain their stemness by directly repressing the expression of *bag of marble* (*bam*; Chen and McKearin 2003; Song *et al*. 2004). After asymmetric cell division, one daughter cell physically associated with the niche cells retains the GSC fate by maintaining BMP signaling activity, while the other daughter cell moves away from the niche cells, losing BMP signaling activity and differentiates into a cystoblast. Consistent with this, the overexpression of Dpp results in a defect in differentiation (a downregulation of *bam*) and causes an accumulation of GSC-like tumor cells in the germarium (Xie and Spradling 1998; Song *et al*. 2004). A second mechanism that limits BMP responses to the GSCs adjacent to the niche is through an inability to restore Mad protein levels in the cystoblast daughter cell, through the action of Brain tumor (Brat)–Pumilio complexes that bind the 3′ UTR of the Mad mRNA to block translation (Harris *et al*. 2011). Interestingly, while it is clear that both BMP ligands are essential niche factors, the overexpression of Gbb influences neither *bam* expression nor GSC maintenance (Song *et al*. 2004). Further studies are required to understand how these ligands cooperatively control the maintenance of the GSC niche.

To ensure short-range BMP signaling, both Dally and Collagen IV tightly regulate extracellular BMP actions (Chen *et al*. 2011; Nakato and Li 2016). *dally* null and hypomorph mutants exhibit no or reduced BMP activity and thus exhibit germaria with fewer or no GSCs (Guo and Wang 2009; Hayashi *et al*. 2009). *dally* is strongly expressed in cap cells, but not in escort cells. Expectedly, exogeneous Dally expression in cap cells is able to rescue the mutant phenotypes, and *dally* RNAi in those cells phenocopies defects in GSC maintenance (Guo and Wang 2009). In cap cells, Dally acts as a BMP trans-coreceptor to promote BMP signaling in GSCs (Guo and Wang 2009; Hayashi *et al*. 2009; Dejima *et al*. 2011). Dally is also thought to control extracellular BMP concentration by either stabilizing or trapping them in the niche region (Guo and Wang 2009; Hayashi *et al*. 2009). Consistent with this idea, Dally overexpression in escort cells abnormally activates BMP signaling in the germarium and blocks GSC differentiation, thus resulting in GSC hyperplasia similar to the Dpp overexpression phenotype (Guo and Wang 2009; Hayashi *et al*. 2009). Likewise, ectopic transcriptional initiation of *dally* in escort and escort stem cells caused by aberrant epidermal growth factor receptor-mitogen activated protein kinase (EGFR-MAPK) signaling leads to an accumulation of GSC-like cells (Liu *et al*. 2010). In this process, Dally seems to act as a major glypican since *dally dlp* double mutant has no additive effect on BMP signaling and *dlp* RNAi in the cap cells has no effect on GSC maintenance (Guo and Wang 2009; Hayashi *et al*. 2009). It is worth mentioning that in the male GSC niche where Gbb has more profound niche factor activity than Dpp (Kawase *et al*. 2004), Dlp acts as the primary glypican for male GSC maintenance although both glypicans are expressed in niche cells (Hayashi *et al*. 2009). Further, Dlp functions together with Gbb for regulating neuroblast proliferation (Kanai *et al*. 2018). It is of interest when considering the extracellular regulation of BMPS this tissue-dependent glypican selectivity. Consistent with this observation, it is reported that Dally can enhance both Dpp and Gbb signaling, while Dlp only promotes Gbb signaling activity (Dejima *et al*. 2011). Thus, distinct coreceptor activities may reflect a tissue-specific usage of glypicans for precisely controlling BMP signaling in the extracellular space.

Another critical niche component, Collagen IV, is not only found in the basement membrane of germarium but also shows a graded distribution diminishing posteriorly in the niche (Wang *et al*. 2008; Van De Bor *et al*. 2015). Hypomorph mutants of *vkg*, which encodes a subunit of Collagen IV, exhibit an expansion of the BMP action range, leading to excess GSCs in the germarium (Wang *et al*. 2008). Interestingly, Collagen IV proteins are nonautonomously deposited by both adult fat cells and ovarian hemocytes (Van De Bor *et al*. 2015; Weaver and Drummond-Barbosa 2018). Subsequent tissue-specific Collagen IV knockdown by RNAi shows that hemocyte-driven Collagen IV is essential for proper BMP activity, thereby contributing to GSC homeostasis (Van De Bor *et al*. 2015). A recent study reveals that adult fat cell–derived Collagen IV is also required for GSC self-renewal by maintaining normal E-Cadherin levels via β-integrin signaling (Weaver and Drummond-Barbosa 2018). Intriguingly, this distinct pool of Collagen IV does not affect BMP signaling, suggesting that Collagen IV regulates GSC homeostasis in at least 2 different ways. This nontissue autonomous action of Collagen IV is not only observed in the germarium, but it is also widely utilized in other developmental processes. For instance, Collagen IV required for proper BMP signaling in the developing wing disc is supplied by the larval fat body (Pastor-Pareja and Xu 2011; Ma, Cao, *et al*. 2017). Additionally, as in the GSC niche, hemocyte-secreted Collagen IV plays an essential role in the malpighian tubule guidance during embryogenesis (Bunt *et al*. 2010). Hemocytes secrete and deposit Collagen IV on the growing malpighian tubule to enhance sensitivity to a locally acting guidance cue, Dpp, produced by midgut visceral mesoderm, thereby achieving the stereotypic malpighian tubule trajectory.

##### BMPs in intertissue communications

While autocrine and paracrine actions of BMP ligands have been extensively explored, recent findings suggest an additional role of BMPs in intertissue signaling (Li *et al*. 2013; Setiawan *et al*. 2018; Denton *et al*. 2019; Robles-Murguia *et al*. 2020). For instance, Dpp acts as a circulating systemic signal controlling the onset of metamorphosis (Setiawan *et al*. 2018). In addition to its well-studied morphogen function, Dpp proteins originating from the developing wing discs reach the prothoracic gland (PG) to inhibit the biosynthesis of the steroid hormone ecdysone during the early larval stages. Later, as the imaginal discs grow, BMP activity in the PGs diminishes, probably due to the trapping Dpp proteins within the discs. This reduction in BMP activity allows the PG to escalate ecdysone production, thereby triggering pupariation. Another study also posits that Dpp expressed in the larval midgut is secreted into the hemolymph, activates the pathway in PG, and perturbs ecdysone production (Denton *et al*. 2019). Further, it has been demonstrated that Dpp derived from the trachea can control adult midgut homeostasis by regulating intestinal stem cell (ISC) activity (Li *et al*. 2013). Dpp proteins expressed in tracheal cells traverse the visceral muscles and signal enterocytes to protect them from cell death, thereby limiting ISC proliferation. Last, muscle-secreted Dpp ligands regulate adult feeding behaviors by modulating dopamine biosynthesis (Robles-Murguia *et al*. 2020). In this case, Dpp ligands from muscles activate BMP signaling in dopaminergic neurons and control tyrosine hydroxylase expression, the late limiting factor in the dopamine biosynthesis pathway. Looking ahead, future investigations will shed light on the systemic actions of BMPs and will provide a more comprehensive understanding of the signaling properties of BMP ligands as more than paracrine and juxtacrine signaling factors.

Thus, in theory, a small number of BMP-encoding genes can generate a larger set of functionally different ligands through the combinatorial actions of different types of posttranslational regulation, including the formation of different ligand forms via different combinations of dimerization and proteolytic processing, combined with different modifications. In addition to the generation of different ligand types, the presence of different extracellular regulators will influence the distribution and activity of each ligand form. Together, the diversity of active ligands produced will contribute to a range of signaling outputs in disparate biological systems. Given the different signaling capacities of different ligand forms, it is important to be mindful that studies that make use of overexpressed genes most certainly affect the stoichiometry of the different ligand pools and thus the signaling output. The design of experiments to investigate the relative effects of different ligands requires an understanding of such shortcomings of overexpression studies as they will impact the balance between ligand isoforms and will bias our interpretation of the true requirements or mechanistic actions of BMP signaling molecules.

### Regulation of receptor availability and signal transduction

It has been well documented that the function of TGF-β/Activin type I and type II receptors can be altered by various protein modifications (Kang *et al*. 2009), but much less is known about such modifications of BMP receptors. However, the availability of BMP receptors and their signaling potential has benefited from studies in both vertebrates and *Drosophila* (Di Guglielmo *et al*. 2003; Mitchell *et al*. 2004; Hartung *et al*. 2006; Xu *et al*. 2012). The molecular mechanisms controlling BMP receptor localization at the cell surface, their clustering in membrane microdomains, and their trafficking through endocytosis for recycling or degradation are emerging from these studies. Below, we provide several examples to illustrate the range of mechanisms used to regulate receptor availability and signaling competence gleaned from studies in *Drosophila*.

#### Receptor type and availability

##### Level of receptors

While remarkably little is known about the transcriptional regulation of receptor genes, several studies have reported the impact of receptor level on signaling output and the different ways in which it can be controlled. *mir124* is a critical regulator of diurnal activity, and although the direct target of *mir124* is not known, it has been shown that heterozygosity of *tkv*, *sax*, and/or *Mad* can rescue phenotypes associated with a *mir124* mutant (Sun *et al*. 2012; Garaulet *et al*. 2016). These data illustrate that the dosage and likely cellular abundance of downstream BMP signaling components can impact circadian rhythm. As discussed above, GSC maintenance depends on the reception of Dpp and Gbb signals by Tkv, Sax, and Punt expressed in the GSCs (Xie and Spradling 1998; Kawase *et al*. 2004; Song *et al*. 2004). The level of BMP signaling in this context appears to be dependent on *aubergine* (*aub*), as *aub* mutant GSCs exhibit a reduction in BMP signaling activity. Aub is a Piwi-family protein that binds to the 3′ UTR of the Bam mRNA to control its translation and thus block differentiation. Aub has also been shown by iCLIP to bind to the 5′UTR, 3′UTR, or both of *punt* and *tkv* mRNAs, suggesting that they, too, may be regulated at the level of translation, suggesting that the level of receptor protein influences signaling activity (Ma, Zhu, *et al*. 2017).

##### Spatial distribution of receptors

Within the GSC niche, multiple Wnt ligands produced by cap cells regulate tkv expression in stromal cells (Luo *et al*. 2015). Tkv in stromal cells removes excess Dpp, thus limiting the “stemness” of cells in the niche. In the wing imaginal disc, *mtv* is known to repress *tkv* expression in the A/P stripe in response to *en* and *Hh* (Funakoshi *et al*. 2001). The downregulation of *tkv* at the A/P boundary turns out to be essential for the proper establishment of the BMP activity gradient that patterns the wing primordium.

##### Receptor isoforms

Multiple splice forms of type I receptor genes, *tkv* and *sax*, are predicted to generate different protein isoforms, 4 Tkv receptor isoforms, and 3 Sax receptor isoforms (Brummel *et al*. 1994; Penton *et al*. 1994). In all cases, alternative splicing results in differences in the extracellular domain. In addition to the generation of different receptor isoforms that have different protein domains in the extracellular region, the alternative transcriptional sites and 5′ UTR sequences could also influence posttranscriptional regulation, such as temporal or spatial control over translation. Such regulation through the 3′ UTR is unlikely as in both genes all splice forms contain common 3′ ends. While the functional contributions of different Tkv and Sax isoforms to signaling are not yet known, results from studies on Babo, the *Drosophila* activin type I receptor, have provided insight into the possible impacts on signaling output. Studies examining the 3 isoforms of Babo suggest a binding preference for the 3 activin-like ligands, each with a somewhat different outcome (Zhu *et al*. 2008; Jensen *et al*. 2009; Awasaki *et al*. 2011).

Studies in Tv4 neurons indicate the importance of receptor isoform-specific functions where it has been shown that *brr2* (BRR2), a U5 snRNP subunit with helicase activity, is critical for the proper splicing of *tkv* (and *Medea*). The splice form of Tkv produced by Brr2 displays a higher affinity for ligand binding than other isoforms. Brr2-mediated splicing is required to attain sufficiently high levels of Tkv-mediated signaling for Tv4 fate specification and FMRFa expression (Monedero Cobeta *et al*. 2018). While it is not known if the alternative splice forms of Sax alter ligand binding affinity or display functional differences, it has been shown that members of the Elav/Hu family of RNA binding proteins (RBPs), Elav, Rbp9, and Fne, are required in the larval CNS to produce specific splice forms of *sax*, as well as *Medea* and *LimK* (Lee *et al*. 2021).

#### Receptor complex composition

The BMP signaling receptor complex is a heterotetramer composed of 2 type I and 2 type II receptors. Type I is the high-affinity receptor for BMPs. Different binding affinities have been determined for specific BMP ligand–type I receptor pairs, including with *Drosophila* components (Miyazono *et al*. 2010; Nickel and Mueller 2019; Gipson *et al*. 2020). Given the dimeric nature of ligands as well as the contribution of each receptor type, the final ligand–receptor hexameric signaling complex can be composed of a number of different molecular combinations with the most varied, a heterodimeric ligand bound to a complex of heterodimeric type I receptors and heterodimeric type II receptors. For example, in *Drosophila* this complex could consist of Dpp/Gbb, Tkv/Sax, and Punt/Wit. The structure of the ligand, analogous to a left hand, is such that the heel of one monomer, and the fingers of another monomer create the type I binding pocket (Allendorph *et al*. 2006; Ehrlich *et al*. 2011; Hinck *et al*. 2016; Yadin *et al*. 2016). This means that Dpp/Dpp, Gbb/Gbb, and Dpp/Gbb each differ in their type I receptor binding pockets. Furthermore, the 2 type I binding pockets on either side of the Dpp/Gbb heterodimeric ligand are different from one another with respect to the contact residues that affect the affinity of ligand–receptor binding. Thus, it follows that the ligand-to-type I receptor binding affinities are most likely different, not only between homodimeric and heterodimer ligands but that the heterodimeric ligand generates 2 different binding pockets with different affinities for each type I receptor ectodomain. Such differences in affinities have not been measured, yet genetic studies suggest they are likely to affect signaling activity. The type II receptor contacts the ligand in the knuckle region of a monomer, and its binding is likely not altered in a heterodimeric ligand.

It is also important to consider that receptor complexes composed of different combinations of type I and type II receptors are likely to be expressed in the same cell. How the signal initiated by different ligand types is received and transduced by the same or different receptor complexes is not fully understood. Furthermore, the interpretation of “promiscuous” signaling resulting from 2 different homodimers associating with a single receptor variant is also not clear, yet such interactions increase the complexity and combinatorial nature of the BMP signaling pathway. Efforts to model the consequences of such multiligand–receptor interactions in vertebrate BMP systems (Antebi *et al*. 2017; Klumpe *et al*. 2022; Su *et al*. 2022) have not yet been applied specifically to Drosophila contexts; it provides a valuable framework to consider the impact of such interactions on signaling output.

#### Receptor complex signaling competence

Studies in the embryo have shown that Tkv and Sax are both required for DV patterning (Schupbach and Wieschaus 1989; Affolter *et al*. 1994; Brummel *et al*. 1994; Nellen *et al*. 1994; Penton *et al*. 1994; Terracol and Lengyel 1994; Xie *et al*. 1994; Twombly *et al*. 2009), especially for higher levels of signaling elicited by the Dpp/Scw heterodimer (Nguyen *et al*. 1998). In this context, Punt appears to be the sole type II receptor as *wit* mutants show no early embryonic defects (Marqués *et al*. 2002). In the wing imaginal disc, both Tkv and Sax, with Punt, are again responsible for mediating signaling, but in this case elicited by Dpp and Gbb ligand combinations (Brummel *et al*. 1994; Nellen *et al*. 1994; Penton *et al*. 1994; Terracol and Lengyel 1994; Singer *et al*. 1997; Haerry *et al*. 1998; Khalsa *et al*. 1998; Tanimoto *et al*. 2000; Ray and Wharton 2001; Bangi and Wharton 2006b; Bauer *et al*. 2023). At the larval NMJ, Wit, instead of Punt, collaborates with both Tkv and Sax to mediate Gbb signals (McCabe *et al*. 2003; Rawson *et al*. 2003; Marqués and Zhang 2006).

In each case, the requirement for both type I receptors is demonstrated by the observation that loss of *tkv* or *sax* is associated with a decrease in phosphorylation of the Smad signal transducer, Mad (pMad; Tanimoto *et al*. 2000; Dorfman and Shilo 2001; McCabe *et al*. 2003; Rawson *et al*. 2003; Bangi and Wharton 2006b). While both receptors are required to mediate optimal signaling, their functional requirements in different development contexts are not equivalent as evidenced by the differences in their loss of function phenotypes. Genetic rescue experiments of ligand overexpression phenotypes by the coexpression of dominant-negative type I receptors concluded that Sax is the high-affinity receptor for Gbb and Tkv is the high-affinity receptor for Dpp (Haerry *et al*. 1998; Nguyen *et al*. 1998). However, loss of function studies showed that *gbb* mutant phenotypes do not phenocopy *sax* mutant phenotypes (Singer *et al*. 1997; Khalsa *et al*. 1998; Ray and Wharton 2001), and *gbb* mutants are not enhanced by loss of function alleles of *sax*, but rather by a loss of *tkv*, together suggesting that Sax does not serve as the sole receptor mediating Gbb and that Tkv plays a part in mediating a Gbb signal (Bangi and Wharton 2006b). Curiously, *gbb* hypomorphic phenotypes were shown to be enhanced by the overexpression of wild-type sax, suggesting that an increase in Sax receptors blocks ligand function. These and other data (Le 2014) based on both overexpression and loss of function revealed that the Sax receptor possesses the ability to inhibit BMP-induced signaling. A model emerged proposing that homodimeric Sax receptor complexes (Sax/Sax) are not able to transduce signals (incompetent), while Tkv/Tkv and Tkv/Sax are able (competent) to phosphorylate Mad (Bangi and Wharton 2006b). By extension, the model suggests that the signaling capacity of different ligand pools will be influenced by the presence of Sax/Sax complexes, which bind but fail to transduce a signal (Bangi and Wharton 2006b; Le 2014). A mutation in the GS activation domain of the Sax receptor, analogous to that responsible for the heterotopic bone disease, fibrodysplasia ossificans progressiva, removes the inhibitory nature of Sax (Le *et al*. 2018). Interestingly, mutations in the GS domain of Sax impact its signaling ability only in the presence of the type II receptor. This finding highlights the importance of type I/type II complex formation, as well as the GS domain in activation of the type I receptor in signal transduction.

#### Posttranslational modifications and receptor signaling competence

The signaling competence of type I receptors can also be affected by posttranslational modifications. Modifications of individual receptor types may influence complex formation and/or the activity of the receptor itself. A recent study found that the *Drosophila* O-GlcNac transferase, *super sex combs* (*sxc*), affects BMP signaling in the embryo, and the O-glycosylation state of Sax in embryos depends on *sxc* (Moulton *et al*. 2020). A putative O-glycosylation site, based on an in silico prediction, resides just outside the GS domain. It would be important to know if the addition of O-glycans blocks the ability of the type II kinase from phosphorylating serine residues in the type I GS domain of Sax, providing a potential mechanistic explanation for the inability of Sax to transduce a signal. Interestingly, O-glycosylation has also been shown to regulate cleavage of the Gbb proprotein at the S1/S0 proconvertase cleavage site in 3rd instar larvae but not in S2 cells consistent with observations that O-glycan decoration is context dependent (Anderson and Wharton 2017).

Sax maintains its inhibitory behavior in S2 cells but whether it is O-glycosylated in these cells is not yet known. Further studies that elucidate the O-glycosylation as well as phosphorylation states of Sax and other type I receptors are warranted as such modifications will aid in our understanding of molecular mechanisms underlying the regulation of receptor signaling competency. In a related vein, it is important to note that changes in diet can regulate BMP signaling by not only carbohydrate metabolism (Ghosh and O’Connor 2014; Moulton *et al*. 2020) where it has been suggested that O-glycosylation acts as a sugar sensor (Bond and Hanover 2015) but also by lipid metabolism (Ballard *et al*. 2010; Chatterjee and Perrimon 2021).

Overall, the precise mechanisms by which type II receptors regulate signaling output have been less studied. However, as noted, Wit and Punt contribute to signaling complexes with Tkv and Sax in different developmental contexts in *Drosophila* (Upadhyay *et al*. 2017). Punt is critical for mediating BMP signaling during wing patterning when the Gbb15 isoform appears most prominent in signaling. However, during pupal ecdysis when cleavage to generate Gbb15 is blocked, Wit is the type II receptor critical for mediating the Gbb38 signaling (Anderson and Wharton 2017). Taken together, multiple studies implicate receptor composition and modification, coupled with different receptor complex–ligand associations, and point to the importance of such interactions in generating the diversity of signaling outputs seen in different developmental and tissue contexts.

#### Subcellular compartmentalization of receptors

BMP receptors must be delivered to the plasma membrane for ligand binding to elicit signal transduction. Surprisingly, very little is known about the production and transport of BMP receptors to the plasma membrane. Ligands can act nonautonomously in a paracrine manner or cell-autonomously in an autocrine manner. BMP receptors appear to act solely in a cell-autonomous manner, i.e. there is no evidence of ectodomain shedding, or cleavage, whereby the extracellular domain impacts surrounding cells, with the exception of the transfer of Tkv from GSC MT-nanotubes into testis hub cells for degradation (Ladyzhets *et al*. 2020). However, the localization of BMP receptors to particular membrane compartments has been observed and shown to affect signaling outcome by limiting ligand–receptor interaction, influencing the composition of receptor complex assembly and focusing on active signaling to specific cellular compartments.

*baiser* (*bai*) and *eclair* (*eca*) are essential for dorsoventral patterning in the embryo and encode 2 p24 proteins important in the transport of secreted and transmembrane proteins into plasma membranes (Bartoszewski *et al*. 2004). They are specifically required for maternal Tkv activity and not zygotic Tkv, but how they affect this activity is not known. It is interesting that the maternal Tkv isoform possesses a leader that could allow for interaction with Bai and Eca, although such studies have not been done. No major defects in Tkv abundance or intracellular localization were observed; however, specific colocalization with components of the secretory vs endocytic machinery was not resolved in this study in embryonic cells.

In the pupal wing, Tkv is localized to the basal side of the PCV primordia. The Scrib complex was identified in a screen for factors important in PCV formation, and *scribbled* (*scrib*) was shown to be important for the localization of Tkv to the basal membrane (Gui *et al*. 2016). Furthermore, Scrib facilitates the internalization of Tkv to Rab5 endosomes following ligand–receptor binding enabling the high levels of signaling required for PCV formation (see below). BMP signaling activity in turn upregulates *scrib* transcription creating a positive feedback loop for optimal signaling presumably by ensuring that Tkv is localized to the membrane compartment that yields the highest level of signaling.

In the larval wing imaginal disc, while the type II receptor Punt is enriched in the basolateral membrane, Tkv is not. The Wit type II receptor is found on all membranes and enriched in the apical membrane (Peterson *et al*. 2022). A short juxtamembrane basolateral targeting determinant targets Punt to the basolateral membrane in both *Drosophila* wing discs as well as in mammalian MDCK cells. Basolateral localization of Punt is critical for optimal signaling, as apical targeting of Punt fails to transduce a signal despite a pool of Dpp ligands in the disc lumenal space. Researchers found no evidence that endocytosis played a role in basolateral localization of Punt via the removal of apical Punt, but rather a dependence on the AP-1 adaptor protein, a key mediator of vesicular sorting and membrane trafficking.

As we can see, the compartmentalization of receptors to discrete membrane domains differs between cell types. Unlike the wing disc, both Punt and Wit are localized basolaterally in the salivary gland. In the follicular epithelium of the egg chamber, Punt and Wit are found uniformly distributed in the apical and basolateral membranes. The variation in receptor localization to discrete domains prompts us to ask more specifically how the compartmentalization of type I receptors, as well as type II, impact their ability to access ligands. Furthermore, it will be important to better understand the functional implications on the level and duration of signaling, of targeting receptors to defined membrane domains. In one of the most extreme cases, Tkv was found to be localized to a fine protrusion of a cell, i.e. a cytoneme in wing disc cells (Roy *et al*. 2011, 2014; Casas-Tinto and Portela 2019) or a MT-nanotube in GSCs (Inaba *et al*. 2015; Ladyzhets *et al*. 2020). In both cases, if the specialized cellular structure is disrupted, BMP signaling is compromised. In the GSC niche, MT-nanotubes ensure the delivery of the Tkv receptor to the ligand-producing hub cell, which allows for precise short-range signaling between the hub and the GSC. In addition, the internalization of Tkv by the hub cell from the GSC MT-nanotube serves to regulate the level of BMP receptor available for signaling (Ladyzhets *et al*. 2020).

#### Regulation of receptor stability and trafficking

##### Receptor stability

Engagement of secreted BMP ligands with the ectodomains of type I and type II receptors results in phosphorylation of serine residues in the type I GS domain by the constitutively active type II S/T kinase, thereby activating the type I S/T kinase. It is thought that dephosphorylation of the activated type I GS domain could be a point of downregulation of the pathway. A yeast 2-hybrid screen for the protein phosphatase PP1c, or *flap wing* (*flw*), revealed an interaction with Sara, Smad anchor for receptor interaction (Bennett and Alphey 2002). A mutation in SaraF678A disrupts binding with PP1c and exhibits phenotypes consistent with elevated levels of BMP signaling in wings. The Sara mutation also leads to hyperphosphorylation of the TGF-β type I receptor in mammalian cells, consistent with the idea that PP1c may normally act as a negative regulator of BMP signaling. Further studies in other contexts are needed to clarify the universality of type I receptor dephosphorylation as a regulatory mechanism.

In addition to “deactivating” type I receptors, several factors have also been identified that affect its degradation. Ribosomal protein S6 kinase-like (S6KL) and the S/T kinase Fused (Fu/Smurf) have been shown to interact with Tkv in vitro and influence its degradation (Xia *et al*. 2010; Zhao *et al*. 2015), while Neuroligin 4 (Nlg4), also known to physically interact with Tkv, instead appears to stabilize the receptor at the presynaptic membrane by inhibiting the action of these kinases, by an as-yet-unknown mechanism (Zhang *et al*. 2017). Ube3A E3 ubiquitin ligase Ube3A specifically ubiquitinates Tkv, not Sax or Wit, in the cytoplasmic domain, promoting proteasomal degradation of Tkv (Li *et al*. 2016). The demonstration that *ube3A* mutants exhibit hyperactivation of BMP signaling at the *Drosophila* NMJ is of particular interest as Ube3A is associated with neurodevelopmental defects in Angelman syndrome and autism.

Once phosphorylated by the type II kinase, the activated type I kinase phosphorylates the R-Smad, activating it for entry into the nucleus where it acts as a transcriptional regulator. There is remarkably little molecular understanding of the specific events leading to the activation of the BMP type I receptor kinase; however, how the activated receptor complex is regulated is starting to take shape. It has been shown in vertebrate cells that type I and type II BMP receptors are continuously endocytosed via clathrin-coated pits, with evidence that type II receptors can also make use of caveolae (Hartung *et al*. 2006). The cytoplasmic tail of different BMP type II receptors may dictate alternative routes of endocytosis (Amsalem *et al*. 2016). Such different modes of internalization have been correlated with Smad-dependent versus Smad-independent signaling, although the stoichiometry of the type I and type II receptors between the different compartments suggests that another means of internalization is likely (Bragdon *et al*. 2011).

##### Receptor trafficking: downregulation versus enhancement of signaling

**Fig. 3.**
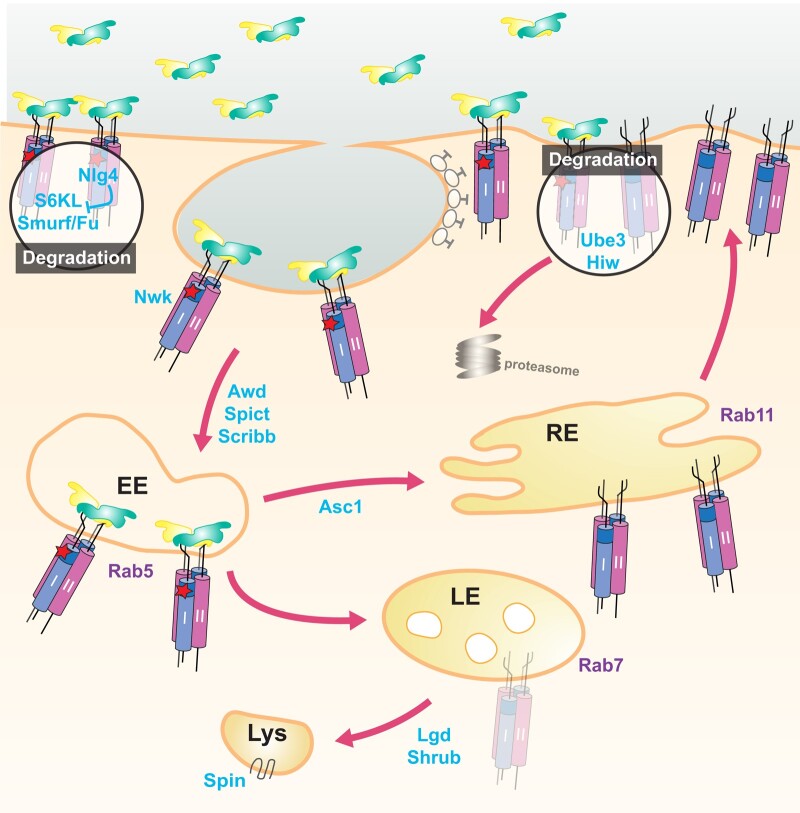
Receptor trafficking and degradation. BMP ligands interact with the ectodomain of type I and type II receptors in the extracellular space. Upon binding and activation of the type I receptor (star), the cytoplasmic Mad protein is phosphorylated prior to localizing to the nucleus where it regulates transcription (not shown). The ligand/receptor complex can be trafficked through different routes, first via clathrin-mediated or caveolae-mediated endocytosis. Nwk is thought to facilitate this process by binding to both Tkv and dynamin. Awd, Spict, and Scribb influence trafficking to the early endosome (EE) marked by Rab5 where active signaling has been observed. Asc1 facilitates trafficking to the recycling endosome (RE) marked by Rab11, from which receptors are thought to be delivered back to the cell surface. Alternatively, the receptor complex enters the late endosomal compartment (LE) destined for the lysosome (Lys) marked by Spin, trafficking mediated in part by Lgd and Shrub. Ube3 and Hiw, E3 ubiquitin ligases, have both been shown to target receptors to the proteosome. Ube3 preferentially enhances degradation of Tkv and not Sax or Wit. Nlg4 binds Tkv and prevents the action of S6KL and Smurf/Fu, both of whom have been shown to increase the degradation of Tkv.

Endocytosis, in general terms, is thought to be a means by which signaling pathways are downregulated, including BMP signaling (Fig. 3). In adult ovarian germ cells, aberrant Tkv trafficking is associated with ectopic BMP signaling activity (Morawa *et al*. 2015). During oogenesis, *lethal (2) giant discs* (*lgd*) mutant germ cells accumulate Tkv in mature endosomes due in part to a failure in the degradation of this transmembrane receptor. The disruption in Tkv trafficking is linked to Shrub, a fundamental component of the ESCRT trafficking machinery, which physically interacts with Lgd. Thus, it appears that wild-type trafficking of Tkv from early to mature endosomes and eventual fusion with lysosomes is regulated by Lgd and the ESCRT-III core component, Shrub, in germ cells. Similarly, BMP signaling at the NMJ was shown to be attenuated by *spinster*, a multipass transmembrane protein that localizes to the lysosome (Sweeney and Davis 2002). Mutations in *spin* result in synaptic overgrowth attributed to an increase in BMP signaling, suggesting that degradation of active receptors is blocked by the loss of Spin in the late endosomal/lysosomal compartment. However, data to the contrary have also been reported that clearly demonstrate that internalization and trafficking of receptors to the Rab5+ early endosome enhances BMP signaling output and in some cases is thought to be required for signaling. From early endosomes, receptors are either shuttled to Rab11+ recycling endosomes and sent back to the cell surface or to late endosomes and targeted for the lysosome and degradation. For the most part, our understanding of receptor dynamics and trafficking is based on biochemical studies in mammalian cells and in some cases verified in *Drosophila* systems (Bokel *et al*. 2006; Chen 2009; Amsalem *et al*. 2016; Deshpande and Rodal 2016; Ehrlich 2016). However, a number of genetic and in vivo studies have uncovered regulators of receptor stability and endocytosis. Mutant interactions and experiments making use of tagged receptors and other signaling components to visualize trafficking in vivo have revealed the importance of endocytosis in BMP signaling regulation (reviewed in Deshpande and Rodal 2016). Some of these results are outlined below and show that regulation of signaling complex internalization and trafficking can occur at multiple points, in some cases boosting signaling while in others downregulating signaling. While it is not yet clear if the different response is cell type specific, the work being done in *Drosophila*, in an in vivo context, is sure to advance our knowledge of how receptors can be regulated and illustrates the context-dependent nature of such regulation.

Most studies have focused on Tkv trafficking and shown that it is controlled by a variety of genes that act at different points in the endocytic process. In a number of cases, internalization is thought to remove Tkv from the cell surface, preventing ligand binding and downregulating the pathway, while in other cases, endocytosis into early endosome (Rab5+) is required for maximal signaling, for segregation into daughter cells, and/or for axonal transport of receptors to the neuronal cell body (Bokel *et al*. 2006; Smith *et al*. 2012). Much of our understanding of the impact of endocytosis on BMP signaling activity has come from studies at the larval NMJ as discussed below and reviewed in (Marqués and Zhang 2006; Bayat *et al*. 2011; Vicidomini and Serpe 2022).

Dynamin is required for clathrin-mediated endocytosis and thus for endocytosis of BMP receptors. *Nervous wreck* (*nwk*) mutants show an increase in the number of synaptic boutons and an elevation of pMad within the synapse, indicating that Nwk normally downregulates BMP signaling (O’Connor-Giles *et al*. 2008). Nwk physically interacts with Tkv, with dynamin, and with dap160, components of the endocytic machinery, suggesting that endocytosis attenuates Tkv-mediated BMP signaling. Consistent with a role for endocytosis as a means to reduce BMP signaling, loss of omega2-adaptin, which normally associates with clathrin to mediate endocytosis, results in an increase in Tkv receptors at the presynaptic membrane and in early endosomes, with a concomitant increase in BMP signaling and synaptic growth (Choudhury *et al*. 2022). While a loss of omega2-adaptin would lead to a reduction in clathrin-mediated endocytosis, the authors point out that Rab11 is reduced in omega2-adaptin mutants, and the observed increase in BMP signaling could reflect a failure of Tkv to be trafficked into recycling endosomes.

Indeed, the balance between directing receptors to the recycling endosome or to the late endosomal/lysosomal compartment has profound outcomes on levels of signaling. A study of dAcs1, the *Drosophila* ortholog of acyl-CoA synthetase, concludes that dAsc1 controls the level of BMP signaling via endocytic recycling (Liu *et al*. 2014). The impact of dAsc1 seems to be specific for the activated Tkv receptor as there are no changes in the overall levels of Gbb or Tkv, indicating that dAsc1 is not promoting degradation nor a general effect on trafficking, as synaptic vesicles are unaltered. dAsc1 appears to specifically affect the targeting of active Tkv to recycling endosomes (Rab11) to maintain moderate levels of signaling in early endosomes. *dAsc1* mutants exhibit elevated BMP signaling and synaptic overgrowth. Disruptions in trafficking Tkv from early to recycling endosomes were also observed when modeling ALS by overexpression of hTDP-43 in motor neurons (Deshpande *et al*. 2016). In these larvae, a reduction in synaptic boutons appears to be due to an increase in Tkv trafficking in the recycling endosome, which abnormally attenuates BMP signaling and leads to motor dysfunction.

Spichthyin (Spict) is an early endosome-associated protein that negatively regulates synaptic growth. Spict also acts to downregulate BMP signaling at the NMJ (Wang *et al*. 2007). Spict coimmunoprecipitated with Wit and appears to drive it into Rab5 early endosomes. This is different from the effect of trafficking receptors from early endosome to LE for degradation (Sweeney and Davis 2002). In *spict* mutants, Wit levels in boutons are higher and BMP signaling can be affected by Spict in S2 cells with no change in the level of receptors. It is possible that Spict internalizes “vacant” receptors, so they cannot interact with ligands. It is also possible that Spict targets BMP ligand–receptor complexes to a specific endocytic compartment where they can then signal as has been observed for Notch signaling (Lu and Bilder 2005; Thompson *et al*. 2005).

The regenerative response mounted by ISCs is characterized by 2 phases that involve Tkv differently (Tracy Cai *et al*. 2019). During homeostasis, Tkv levels are kept low via the E3 ubiquitin ligase *highwire (hiw)*–facilitated proteasome-mediated degradation. Following infection and injury of the intestinal epithelium, with Tkv levels reduced, Dpp appears to interact with Sax/Punt to activate Smox/dSmad2, thus inducing Smox target genes that are known to be important for ISC proliferation. During recovery, Tkv levels appear to be stabilized by proteasome inhibition. Elevated levels of a nucleoside diphosphate kinase, AWD (in response to JNK signaling), increase the internalization of Tkv signaling complexes to Rab^+^ early endosomes, promoting pMad-mediated signaling activity (Tracy Cai *et al*. 2019). Interestingly, the authors show that Tkv accumulation at the plasma membrane is not sufficient to activate Mad, but that Mad phosphorylation depends on Rab5 and dynamin-mediated endocytosis. A similar observation made in the pupal wing disc at the PCV shows that a scaffolding protein encoded by *scribble (scrib)* regulates the localization of Tkv to the basolateral membrane and facilitates its internalization into Rab5 early endosomes where Tkv actively signals (Gui *et al*. 2016). Thus, a number of molecular mechanisms have been identified that impact not only receptor trafficking but also the consequences of trafficking on signaling output. At present, we have little information on how different ligand–receptor combinations influence trafficking and degradation and on studies exploring the importance of this form of receptor regulation.

#### Interacting intracellular proteins and Smad-independent signaling

The cytoplasmic domain of the type II receptor is not only required for its localization to specific membrane compartments, but it also mediates Smad-independent signaling. In response to Gbb signals at the synapse, LimK associates with the C-terminal domain of Wit to regulate actin dynamics in the presynapse, critical for synaptic stability and bouton budding (Eaton and Davis 2005; Piccioli and Littleton 2014). LimK-dependent signaling appears to define a distinct branch of BMP signaling that acts locally in its stabilization of the synapse through its ability to deactivate the actin-depolymerizing protein, cofilin. The LimK binding domain in the C-terminus of Wit does not play a role in synaptic growth, a process requiring pMad transport to the motor neuron soma and nuclear localization of pMad to regulate transcription. LimK/BMPRII interactions in mammalian cells and their impact on the regulation of actin dynamics demonstrate that this form of receptor regulator is functionally conserved (Foletta *et al*. 2003). Several other cytoplasmic regulatory proteins are known to bind the intracellular domain of BMP type II receptors in mammals, but they have not yet been tested for a conserved function in flies (Miyazono *et al*. 2010).

Wit and Sax/Tkv are involved in another process at the NMJ that results in pMad accumulation at the presynapse, albeit in this case not initiated by the Gbb ligand (Sulkowski *et al*. 2016). The accumulation of a presynaptic pool of pMad is promoted by GluRIIA, which leads to clustering of GluRs, GluRIIa, and GluRIIB, postsynaptically. A GluR auxiliary protein, Neto, is proposed to link the GluR clustered tetramer on the postsynaptic membrane with BMP receptors on the presynaptic membrane. Synaptic pMad appears to be a sensor of synaptic activity and has no role in regulating synaptic growth (Vicidomini and Serpe 2022). It is not yet known how this pool of synaptic pMad is generated. Is there a ligand other than Gbb, which acts to stimulate the phosphorylation of Mad? What is the exact composition of the BMP signaling complex responsible for phosphorylating Mad? Why is this pool of pMad not transported to the nucleus to engage in transcription? Further studies elucidating the role of BMP receptors in this form of local signaling observed at the NMJ, and testing for similar localized cytoplasmic pools of pMad in other cellular contexts, will be critical for understanding the intricate regulatory mechanisms that enable BMP signaling to coordinate distinct but related cellular processes such as synaptic growth and synaptic activity.

### Regulation of Smads

#### Regulation of Smad nuclear accumulation by C-terminal Mad phosphorylation

Smads are transcriptional regulators, but their presence in the nucleus is controlled by their phosphorylation states. Endogenous Mad and transgenic tagged Mad are each detected predominantly in the cytoplasm of *Drosophila* tissues (Newfeld *et al*. 1997). In both cases, BMP-regulated changes in nuclear localization were obscured at the protein level, except in the presence of overexpressed Dpp. Like vertebrate Smads, Medea can associate with C-terminally phosphorylated Mad (pMad) and accumulates in the nucleus in a pMad-dependent manner (Das *et al*. 1998; Wisotzkey *et al*. 1998). In most tissues, at sites of endogenous BMP signaling, the subcellular localization of Medea appears uniform within the cell (Sutherland *et al*. 2003). However, during early embryonic development, the high BMP activity at the dorsal midline is associated with detectable nuclear accumulation of Medea protein. Antibodies that detect C-terminally phosphorylated Mad (p^C-ter^Mad) reveal nuclear Mad at sites of known BMP signaling activity (Figs 1b and 5a; Eldar *et al*. 2002). From here on, we will use p^C-ter^Mad instead of pMad, to distinguish activation of Mad at the terminal residues versus other sites of phosphorylation in the Mad protein.

Both R-Smads and co-Smads move in and out of the nucleus in the absence of a BMP signal (Pierreux *et al*. 2000; Fig. 5a), however, the dynamics of nuclear accumulation for both Smads is altered once the R-Smad is phosphorylated by the activated type I receptor (Schmierer *et al*. 2008). Evidence from mammalian systems supports a dynamic system of R-Smad phosphorylation by cell surface receptors, which increases accumulation in the nucleus where they may bind DNA or be dephosphorylated. Dephosphorylation appears to accelerate nuclear export; when returned to the cytoplasm, R-Smads may be phosphorylated again if receptors remain activated (Schmierer and Hill 2005, 2007; Schmierer *et al*. 2008). It is thought that cycling R-Smads out of the nucleus in this way gives continuous sensing for receptor activity and thus confers the exquisite sensitivity of BMP responses to differing levels and duration of the extracellular signal.

### Role of phosphatases in R-Smad activity

Three *Drosophila* phosphatases have been demonstrated to remove C-terminal phosphates from pMad (Fig. 5a); all show a similar function in mammalian cell lines. Each has been tested in vivo, by genetic interaction assays to assess patterning in wing imaginal discs or vein formation in pupal wings. For example, the first p^C-ter^Mad phosphatase identified was pyruvate dehydrogenase phosphatase (PDP), which is localized in the nucleus. RNA interference targeting PDP led to an increase in p^C-ter^Mad levels in S2 cells expressing Flag-Mad in the presence of 10^−9^ M Dpp (Chen *et al*. 2006). Similarly, embryos mutant for *pdp* display elevated staining for p^C-ter^Mad. In contrast, when PDP is overexpressed, a Dpp-responsive reporter *Ubx-lacZ* shows decreased expression.

A cytoplasmic and organellar dual specificity phosphatase, myotubularin-related protein 4 (MTMR4), also appears to regulate *Drosophila* BMP signaling (Yu *et al*. 2013). Overexpression of human MTMR4 in S2 cells can accelerate dephosphorylation of endogenous p^C-ter^Mad following Dpp stimulation. In flies, overexpression of human MTMR4 mildly enhances the *vg-Gal4*–driven knockdown of Tkv by in vivo RNAi, as assessed by wing vein morphology. A gene fragment from *Drosophila* CG3632, predicted to be orthologous to MTMR4, was similarly expressed in a UAS-transgene and shown to generate a similar mild enhancement of vein phenotypes typically associated with knockdown of Tkv. Conversely, both human and fly MTMR4 overexpression can mildly abrogate wing vein defects caused by Gbb overexpression. MTMR4 could be involved in the general cytoplasmic quenching of p^C-ter^Mad activity, independent of nuclear import/export.

The third phosphatase, Dullard, has more complex effects on Mad function. Dullard is associated with the nuclear envelope, similar to its yeast homolog, Nuclear Envelope Morphology protein 1 (NEM1; Liu *et al*. 2011). *Drosophila* males hemizygous for *Dullard* (*Dd*) exhibit ectopic wing vein formation that is suppressed by heterozygosity for *tkv*, *sax*, *or punt*. Furthermore, overexpression of *UAS-Dd* alters the wing disc spatial expression domains for *Dad-lacZ* and *brk-lacZ*, established by BMP target gene reporters. Finally, p^C-ter^Mad in testis GSCs is increased in hemizygous *dd* males and reduced with *dd* overexpression. These observations are consistent with data from S2 cells showing that Dullard can dephosphorylate p^C-ter^Mad (Urrutia *et al*. 2016). As discussed below, Dullard also removes phosphates from the linker region of Mad.

Removal of C-terminal phosphates is a potent block to BMP-mediated gene expression, but little work has compared the relative contributions of PDP, MTMR4, and Dullard. In the absence of *Drosophila* cell type-specific biochemical assays that would distinguish contributions of each phosphatase, sensitive assays for levels of p^C-ter^Mad appear useful. Such assays uncovered the role of Dullard in the dephosphorylation of p^C-ter^Mad at the nuclear pore of ovarian GSCs and cystoblasts, as a critical factor in generating asymmetric partitioning of p^C-ter^Mad between the stem cells and their daughters (Sardi *et al*. 2021).

It is important to remember that most phosphatases target multiple phosphoproteins. Dullard exemplifies this tangle in assessing genetic interaction phenotypes to understand how this phosphatase impacts BMP signaling. Orthologs of Dullard in other eukaryotes, including human C-terminal domain nuclear envelope phosphatase I (CTDNEP), yeast Nem1p, and *C. elegans* CNEP1, are all implicated in the dephosphorylation of the phosphatidic acid phosphatase, Lipin, thus regulating nuclear envelope identity and nuclear pore complex biogenesis (Bahmanyar and Schlieker 2020). Dullard overexpression in wing discs is associated with the aberrant distribution of nuclear transporters RanGap and Importin-beta at the nuclear envelope. Knockdown of *Dmel Lipin* by in vivo RNAi produced mild ectopic wing venation phenotypes, ameliorated in a *Dd* hemizygote or by the downregulation of BMP signaling via cooverexpression of Dad (Liu *et al*. 2011), suggesting that a second target of Dd may have independent effects on BMP signaling outputs. Further studies to decipher the potential web of Dullard nuclear envelope–BMP signaling interactions will be required to fully understand how Dullard attenuates BMP signaling in vivo.

#### Impact of linker phosphorylation on Mad function

**Fig. 4.**
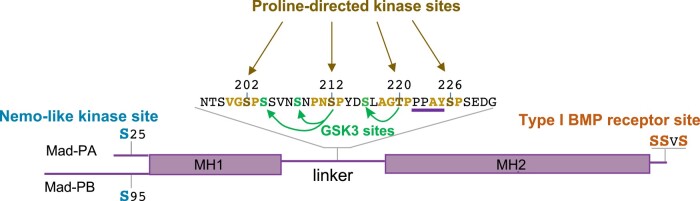
Selected target sites for regulation of Mad activity and localization. All R-Smads and co-Smads share homology in 2 domains: MH1 and MH2. Mad has 2 N-terminal splice variants, conferring distinct N-terminal protein domains, shown here with the shorter Mad-PA N-terminus at the top left of MH1, and the longer Mad-PB N-terminus at the bottom left of MH1 (Sekelsky *et al*. 1995; Wiersdorff *et al*. 1996). The shorter isoform, Mad-PA, is commonly used for transgenic constructs, so the linker region amino acids are indicated by their location in Mad-PA. The linker region lies between MH1 and MH2 and has variable length across animal species, and only small stretches next to MH2 show sequence conservation between Mad and mammalian R-Smads. The MH1 domain contains the DNA binding site; the MH2 domain is involved in Smad–Smad association after phosphorylation. Stronger conservation is found within the R-Smads or within the co-Smads, which recognize distinct DNA binding sites. Inhibitory Smads share the MH2 domain but are divergent in the MH1 region (Hariharan and Pillai 2008). Known sites for the regulation of Mad activity mentioned herein are indicated as follows: Nemo-like kinase phosphorylation sites, S25 in Mad-PA or S95 in Mad-PB, are depicted in the N-terminal region. A proline-/serine-rich 34 amino acid sequence from the linker region is detailed. This short sequence contains 4 sites for proline-directed S/T kinase phosphorylation at 202, 212, 220 and 226. Among these, only phosphorylation at S212 has been studied in detail, but the proximity of additional sites raises the possibility that secondary sites could be targeted alternatively. Several of these sites can direct the kinase GSK3 (Sgg or Zw3 in Drosophila) to phosphorylate a nearby, more N-terminal serine. Linker phosphorylation is thought to recruit the binding of Smurf ubiquitylase to the nearby PPAY motif (underlined). The short C-terminal motif for activated BMP type I receptor phosphorylation is indicated as SSvS.

The duration of the nuclear response to BMP signals can also be regulated by the phosphorylation/dephosphorylation of residues in the linker between the MH1 and MH2 domains of BMP R-Smads (Fig. 4). Such regulation of signaling activity through site-specific phosphorylation of distinct residues in the R-Smad linker has been described (Aragon *et al*. 2011). The original studies suggesting regulation of signaling output via a complex series of differential phosphorylation at discrete sites in R-Smad were performed in mammalian cells during growth and tissue self-renewal. Detailed studies in zebrafish and Drosophila have not yet revealed the full complexity of such a regulatory mechanism in in vivo physiological contexts. Nevertheless, 4 sites for phosphorylation by proline-directed serine-threonine kinases (P_/IVLA_S/TP motif) have been predicted in the *Drosophila* Mad linker domain by PhosphoBase 2.0 (Kreegipuu *et al*. 1999; Fig. 4). To better frame the results indicative of linker phosphorylation of Mad, it is useful to dive into the evidence for linker phosphorylation of its mammalian homolog, Smad1.

##### Smad1 linker phosphorylation

Phosphorylation of residues within the Smad1 linker domain by ERK MAP kinase was first documented in both *Xenopus* embryos and mammalian cultured cells (Kretzschmar *et al*. 1997; Fuentealba *et al*. 2007; Sapkota *et al*. 2007; Chen and Wang 2009; Eivers *et al*. 2009; Gaarenstroom and Hill 2014). These studies found that ERK-directed linker phosphorylation primes subsequent phosphorylation by glycogen synthase kinase 3 (GSK3), which targets a Serine, 4 residues N-terminal to a phospho-S or phospho-T (Cohen and Frame 2001; Fig. 4). This combination of events targets linker-phosphorylated Smad1 (^pLink^Smad1) for proteolytic degradation through the recruitment of the Smurf E3 ubiquitin ligase to a nearby PPAY binding site (Kretzschmar *et al*. 1997; Fuentealba *et al*. 2007; Sapkota *et al*. 2007; Alarcon *et al*. 2009). In the cellular contexts examined, ERK MAP kinase thus antagonizes BMP signaling activity by downregulating the level of transcription mediated by Smad1.

**Fig. 5.**
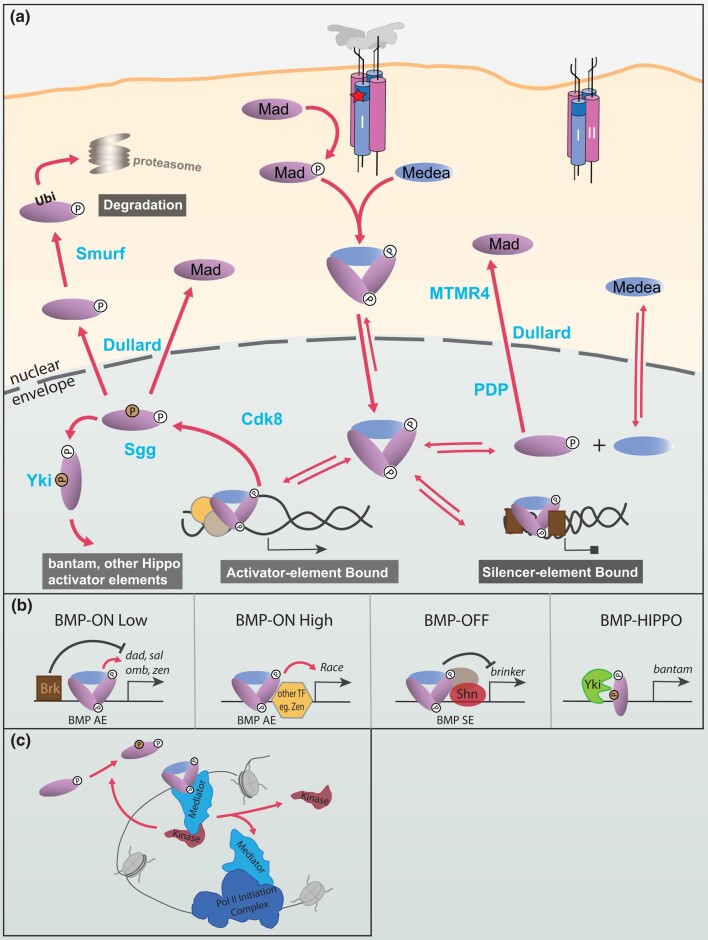
Regulation of Mad. Mad activity is regulated at the level of phosphorylation, nuclear-cytoplasmic shuttling, in association with transcriptional cofactors, as well as the mediator complex. a) Ligand–receptor interaction results in the phosphorylation of C-terminal serines. Two p^Cter^Mad associate with Medea, translocating to the nucleus to either activate or repress transcription. p^Cter^Mad is dephosphorylated by at least 3 phosphatases, PDP, MTMR4, and Dullard, which are each localized to different cellular compartments, nuclear, cytoplasmic, and associated with the nuclear envelope, respectively. Medea cycles in and out of the nucleus but is more likely to be retained when complexed with p^Cter^Mad. Mad is also phosphorylated by other kinases in its linker domain (Sgg and others not shown). Linker phosphorylation recruits Smurf, which facilitates ubiqutination and subsequent degradation of Mad. Linker phosphorylation also mediates association with Yki leading to different transcriptional outcomes. b) Sveral different BMP response elements have been identified, each mediating a different transcriptional response [BMP-AE, BMP silencer element (BMP-SE)]. c) Cdk8 is a component of the mediator kinase module and able to phosphorylate Smad1 and Mad linker domains. Mad and Smad1 are thought to integrate with Cdk8 and the mediator complex to influence transcription.

Subsequent studies showed that phosphorylation of the Smad1 linker by Cdk8/Cdk9 slightly prolonged the nuclear lifetime of p^CTer^Smad1 and promoted the binding of nuclear YAP/TAZ, the transcription factors regulated by Hippo signaling (Alarcon *et al*. 2009). Although an increase in endogenous nuclear p^CTer^Smad1 was observed, mutations in the linker phosphorylation sites actually led to a decrease in target gene expression, presumably because YAP/TAZ binding was blocked. Since Cdk8/Cdk9 are components of the Mediator Kinase Module, which variably associates with the Mediator complex to *activate* transcription (Richter *et al*. 2022; Malik and Roeder 2023; Fig. 5), their effect on R-Smad activity has been called “agonist-induced linker phosphorylation” to distinguish it from the negative impact associated with ERK MAP kinase-mediated phosphorylation which leads to proteolytic degradation of Smad1. Both Cdk8/Cdk9 and ERK MAP kinase phosphorylate sites in the linker of p^CTer^Smad, the BMPs or TGFβ-activated R-Smad (Gao *et al*. 2009). Each kinase binds a docking site (Kliche and Ivarsson 2022) prior to phosphorylating one of the P_/IVLA_S/TP sites in the linker. Notably, phosphorylation by Cdk8/Cdk9 occurs during or immediately subsequent to the initiation of RNA polymerase II transcription, and thus, this kinase acts on R-Smads assembled at transcriptional activation sites.

Alarcon et al. (2009) proposed that distinct WW domain proteins could be recruited in response to different linker phosphoserines based on the action of either ERK or Cdk8/9 kinases (Chen and Wang 2009), with the resultant phosphoS or phosphoT promoting binding to a PPxY motif (Salah *et al*. 2012) and PPAY in Mad (Fig. 4). Thus, class I WW domain proteins can participate in “agonist-induced” as well as in “antagonist-induced” regulation of Smad-mediated BMP signaling. Overall, WW domain proteins have a diverse range of functions including HECT domain type3 ubiquitin ligases, such as Smurf (Chong *et al*. 2006), and the mammalian Hippo pathway-regulated transcription factor YAP (Chen and Sudol 1995), homologous to Drosophila Yorkie (Yki; Huang *et al*. 2005). The Smad phosphocode model proposes that depending on the first linker Serine or Threonine that is phosphorylated, either Cdk8/9 or ERK MAP kinase can trigger different outcomes: YAP/TAZ binding or HECT domain ubiquitin ligase-directed proteolysis (Smurf for Smad 1 and Nedd4 for Smad2/3; Alarcon *et al*. 2009; Gao *et al*. 2009; Aragon *et al*. 2011). Other class I WW domain proteins that similarly bind to the PPxY motif in R-Smad linkers will likely be identified, increasing the number of players able to regulate R-Smad activity through a relatively small number of residues in the linker domain.

##### Mad linker phosphorylation

Of the 4 Mad linker P_/IVLA_S/TP motif sites, only Mad^S212^ has been investigated as the only “canonical” PxSP binding site for ERK MAP kinase (Eivers *et al*. 2011). A *mad* transgene mutant at this site, Mad^S212A^ (Mad^MMM^), greatly reduces polyubiquitylation in human 293T cells compared with a Mad^WT^ transgene, based on Western analysis. Phosphorylation at Mad^S212^ is predicted to prime for GSK3 phosphorylation at 2 sites, Mad^S204^ and Mad^S208^ (Fig. 4). The Drosophila GSK3 gene is *zw3* (*zeste-white 3*), now called *sgg* (*shaggy*; Fig. 5a). A transgene harboring mutations in these 2 GSK3 sites, Mad^S204A,S208A^ (Mad^MGM^), block polyubiquitination in 293T cells to a similar extent as does Mad^MMM^. Consistent with a failure to downregulate Mad-mediated signaling when Mad^MGM^ expression is Gal4-UAS–driven in Drosophila tissues, an expansion in the domain of BMP target reporter genes (*spalt-lacZ* and *omb-LacZ*) is observed compared with when Mad^WT^ transgene is Gal4-UAS–driven. Similarly, knockdown of early embryonic *zw3/sgg* levels leads to expansion of the anti-p^CTer^Mad immunostaining domain, with associated in vivo defects in wing disc patterning and growth (Aleman *et al*. 2014). Overall, these observations parallel those from mammalian cells.

Curiously, in S2 cells, Rolled MAP kinase is not able to phosphorylate Mad^S212^ (Aleman *et al*. 2014), and in third instar larval wing discs, the knockdown of neither Rolled MAP kinase nor dERK2 MAP kinase alone alters *sal-lacZ* expression (Li *et al*. 2020). It remains to be determined whether both *Drosophila* ERK MAP kinases must be knocked out to have an impact on Mad target gene expression. Unlike Rolled MAP kinase, Cdk8 is able to phosphorylate Mad^S212^ in S2 cells (Aleman *et al*. 2014). Furthermore, RNA interference-mediated knockdown of Cdk8 in S2 cells significantly reduces the levels of p^CTer^Mad, as discerned by Western blot analysis with anti-pSmad1/5/8 (Persson *et al*. 1998) consistent with the ability of Cdk8/9 to extend the nuclear lifetime of p^CTer^Smad1 (Alarcon *et al*. 2009). Again, consistent with Cdk8 acting as an agonist, the knockdown of endogenous expression for either Cdk8 or its partner, Cyclin C, in 3rd instar larval wing discs results in a major reduction in the expression of *sal-lacZ* (Li *et al*. 2020). However, while RNAi knockdown of Cdk8 resulted in reduced expression of the *sal-lacZ* reporter, endogenous levels of p^CTer^Mad were unaffected as assayed by immunostaining with the cross-reacting commercial anti–phospho-Smad3 (Abcam ab118825). Despite the alteration in transcriptional response, the inability to detect a change in endogenous p^CTer^Mad levels in situ could reflect the differential sensitivity of Western blots versus immunostaining. Alternatively, the different results could be attributed to the antibodies used or to the differences in the signaling context between S2 cells and imaginal discs.

The major substrate for Cdk-mediated linker phosphorylation is thought to be p^CTer^R-Smads (Chen and Wang 2009) given their greater accessibility when both kinase (Cdk8/Cdk9) and substrate (p^CTer^R-Smad) are at an enhancer–promoter interaction during the activation of RNA polymerase (Fig. 5a). p^S212^p^CTer^Mad appears to be the major substrate for GSK3-mediated phosphorylation at Mad^S204,S208^ in S2 cells, corroborated in vivo by a tight correlation among the domains of anti-p^CTer^Mad immunostaining, anti-p^S212^Mad, and anti-p^S204^p^S208^Mad immunostaining in Drosophila embryos, detected with specific antiphosphopeptide antibodies (Fuentealba *et al*. 2007). Finally, the knockdown of early embryonic GSK3 levels leads to expansion of anti-p^CTer^Mad immunofluorescence (Aleman *et al*. 2014). Whether Cdk8 also phosphorylates Mad^T220^ is unknown.

##### Mad inactivation

It is unclear how nuclear p^S212^p^CTer^Mad becomes available to GSK3. Some studies have reported that GSK3 is associated with centrosomes (Bobinnec *et al*. 2006; Wojcik 2008; Lye *et al*. 2014), although another study has reported a high perinuclear GSK3 localization that correlates with β-catenin destruction (Lybrand *et al*. 2019). It is important to keep in mind that in parallel to the activity of kinase, the multiple phosphorylated R-Smad can be dephosphorylated by one of several phosphatases, sending the R-Smad to the cytoplasm to be recycled for a new interaction with activated receptors, and the question of Kinase accessibility brings us back to 2 phosphatases: nuclear PDP phosphatase and perinuclear Dullard. Phosphatase-mediated dephosphorylation is a conservative mechanism that inactivates Mad for BMP transcriptional responses but preserves the protein for on-going or future signaling responses.

Consistent with the prevailing model for proteolytic degradation of linker-phosphorylated vertebrate Smad1, *Drosophila* Smurf downregulates BMP target gene expression responses in wing and embryonic development (Podos *et al*. 2001; Liang *et al*. 2003; Fig. 5a). In an exogenous assay, Smurf promotes ubiquitylation and degradation of Mad in human 293 cells (Liang *et al*. 2003). However, *Drosophila* Smurf downregulates multiple proteins acting in various signaling pathways, including both Mad and Smox/dSmad2, as well as the BMP type I receptor Tkv (Liang *et al*. 2003; Xia *et al*. 2010). To date, genetic interactions showing that Smurf antagonizes BMP signaling have not been able to directly implicate Mad linker phosphorylation as the mechanism responsible for attenuation of the BMP signal.

It seems likely that linker-directed Smurf-mediated proteolysis is a minor component of endogenous p^CTer^Mad inactivation. Immunostaining indicates that high levels and/or prolonged endogenous BMP signaling are not obviously associated with a decreased pool of cytoplasmic Mad (Newfeld *et al*. 1996). Perhaps specific subcellular pools of p^CTer^Mad are reduced by linker phosphorylation-triggered proteolysis, depending on the availability of GSK3 or Smurf (Gaarenstroom and Hill 2014). Understanding the relative nuclear pools of p^CTer^Mad versus dephosphorylated Mad will be important in specific tissues where Mad has been implicated in a Wg-activated transcriptional complex that incorporates Mad proteins that lack C-terminal phosphates (Zeng *et al*. 2008; Eivers *et al*. 2009, 2011). The importance of Mad–Armadillo–TCF complexes in *Drosophila* tissues is unknown. Wg and BMP signaling show distinct interactions in different tissues at different developmental stages, which may be direct or indirect and may involve additional signaling pathways. In addition to the proposed Mad–Armadillo–TCF complexes, detailed studies of transcription factor responses at specific genes or tissues have uncovered several distinct mechanisms: combinatorial responses integrated at distinct DNA binding sites, physical competition due to overlapping DNA binding sites, cross talk between signal transducers in the cytoplasm or on the centrosome, and super-enhancers that bind nuclear condensates of signal-activated transcription factors (Waltzer *et al*. 2001; Wojcik 2008; Quijano *et al*. 2011; Stroebele and Erives 2016).

#### Phosphorylation impacts subcellular localization

In addition to the effects of linker phosphorylation, the *Drosophila* nemo-like kinase (*nlk*), Nemo, appears to negatively regulate BMP signaling in wing discs, both in terms of wing size and patterning the longitudinal veins (Zeng *et al*. 2007). Nemo phosphorylates Mad at Mad^S25^ of Mad-PA (probably also Mad^S70^ of Mad-PB), adjacent to the highly conserved Mad homology domain I (MH1), when they are coexpressed in HEK293 cells. As such, *nemo* mutants exhibit increased pMad at the NMJ (Merino *et al*. 2009). Studies in human cell lines suggest that coexpression of Nemo with Mad and activated Tkv receptor gives a much lower level of Mad nuclear accumulation than coexpression of Mad with activated Tkv alone (40.1% of transfected cells vs 91.2%, respectively). These data suggested that either p^S25^Mad is normally held in the cytoplasm or Nemo-mediated phosphorylation at Mad^S25^ accelerates nuclear export. A more recent study suggests that phosphorylation of Mad^S25^ regulates the retention of p^CTer^Mad in presynaptic neuronal boutons versus axonal transport back to the nucleus in the cell body (Sulkowski *et al*. 2016).

#### Smad-mediated regulation of gene expression

Smad-mediated regulation of *Drosophila* gene expression has been reviewed elsewhere (Affolter *et al*. 2001); therefore, we will only briefly summarize. Early studies indicated that genes could either be activated or repressed in response to BMP signaling. Smad-binding sites were identified in cis-regulatory DNA for *zerknullt (zen)*, *tinman (tin)*, *labial (lab),* and *vestigial (vg;* Kim *et al*. 1997; Yin *et al*. 1997; Xu *et al*. 1998; Kirkpatrick *et al*. 2001; Gao *et al*. 2005; Xu *et al*. 2005; Gao and Laughon 2007). Notably, a distinct DNA binding site [BMP silencer element (SE)] was identified that recruits a Smad repressor complex, comprised of Mad, Medea, and Schnurri (Marty *et al*. 2000; Pyrowolakis *et al*. 2004; Gao *et al*. 2005; Fig. 5b). The precise spacing of Mad and Medea binding sites within a DNA response element determines whether Schnurri would be recruited to form a repressor complex. Thus, it was determined that Schnurri is essential for BMP-directed gene repression but dispensable for BMP-directed activation of gene expression. These observations resolved the function of Schnurri, which had been implicated in the BMP signal transduction pathway, but did not appear required for all BMP-mediated responses (Arora *et al*. 1995; Grieder *et al*. 1995; Hoodless *et al*. 1996; Torres-Vazquez *et al*. 2000).

Binding sites for BMP-induced activation of expression were more variable, but a conserved BMP-activating element (BMP AE) has also been defined. This work started from the BMP activating enhancer in *dad* and then demonstrated sequence conservation in genes across Drosopholids and BMP response elements conservation in vertebrates. This element binds both Mad and Medea, consistent with other gene's binding sites defined previously.

In the response elements of genes that are activated by BMP signaling, the arrangement of Mad and Medea DNA binding sites varies more broadly. Studies of the *brinker* gene resolved many questions about the mechanisms that limit the spatial extent of expression for a given BMP response gene, in combination with studies of the BMP response elements for other BMP target genes. The wing disc expression pattern for the *brinker* gene attracted attention because its pattern of expression is the inverse of the BMP activity gradient in wing imaginal discs (Minami *et al*. 1999). The finding that Brinker is a transcriptional repressor provided an important key (Jazwinska, Kirov, *et al*. 1999; Jazwinska, Rushlow *et al*. 1999; Xu *et al*. 2005; Weiss *et al*. 2010; Fig. 5b). Direct competition for overlapping Smad and Brinker binding sites appears to occur in only a few genes, including *zen* and *dad*. In other genes, Smad-mediated activation of gene expression is conferred by a separate binding site from the site for Brinker-repression (Barrio and de Celis 2004). Finally, some genes are indirectly activated by BMP signaling through Brinker-mediated repression (Sivasankaran *et al*. 2000). The role of the inverse Brinker repressor gradient in BMP-regulated transcription has been extensively studied in different tissues and is beyond the scope of this review (Campbell and Tomlinson 1999; Marty *et al*. 2000; Sivasankaran *et al*. 2000; Hasson *et al*. 2001; Kirkpatrick *et al*. 2001; Rushlow *et al*. 2001; Saller and Bienz 2001; Zhang *et al*. 2001; Müller *et al*. 2003; Martin *et al*. 2004; Moser and Campbell 2005; Takaesu *et al*. 2008).

Although BMP-activated response genes can be activated at high, low, or moderate levels of BMP signaling, this differential responsiveness rarely appears to reflect variations in the Smad-binding sites (Hamaratoglu *et al*. 2014; Fig. 5b). For genes examined in depth, either the responsiveness to BMP signaling or the complex pattern of signaling is a combinatorial response to multiple signaling pathways (Liang *et al*. 2012; Chayengia *et al*. 2019). In another type of assay, combinatorial signaling between BMP and EGFR signaling has been demonstrated for multiple patterns of reporter gene expression in the ovarian follicle cells (Yakoby, Bristow, *et al*. 2008). Although Tv4 neuron subtype gene expression involves combinatorial regulation with neuron-specific transcription factors, it contains an exceptionally low affinity Mad/Medea binding site (Berndt *et al*. 2020).

A distinct Mad-binding regulatory element was identified for combinatorial signaling by BMP and Hippo signaling pathways (Oh and Irvine 2011; Fig. 5a and b). This element is relevant to linker phosphorylation-mediated Mad activity (see *Impacts of linker phosphorylation on Mad function*), because linker phosphorylation is thought to stimulate the formation of the Mad-Yki complex, through a Smad1/YAP/TAZ complex that generally increases BMP response gene activation (Alarcon *et al*. 2009). Both Mad and the Hippo-pathway controlled transcription factor, Yki, are required for full expression of the microRNA gene *bantam* (*ban*), which negatively regulates cell growth in the wing primordium. Both Mad and Yki activate *ban* expression through a region showing strong activation in a 2.5 kb reporter (*br-2.5).* Mad and Yki physically associate, and their association is facilitated, but not fully dependent upon either the Mad PPAY motif or the Yki WW domain. Medea was not necessary for BMP-induced *br-2.5* expression, making this activating element distinct from the previously described BMP AE. However, Medea is indirectly required for Brk-mediated repression of the same reporter. The canonical Yki DNA binding partner Scalloped was not necessary for this element, but an alternative Yorkie-Homothorax binds an independent site in the same DNA construct. These authors tested whether transgenic activated-Yki could increase the expression of other established BMP target gene reporters, including the *Ubx* DRE reporter in S2 cells (Weiss *et al*. 2010) and *vg*, *omb*, *salr*, and *brk*. Although minor effects on gene expression were observed for *omb* and *vg*, in detail the observations were inconsistent with the activation of expression by endogenous Yki. More recently, Yki knockdown was observed to decrease the expression of the *salm-LacZ* reporter (Li *et al*. 2020), but it is not known whether this effect is direct or indirect. It is not yet clear if the formation of a Yki–Mad complex mediates the expression of only a few specific gene, or if it acts in only some specific physiological contexts. Further studies should provide more clarity.

From a whole-genome perspective, candidate regulatory regions for different modes of BMP regulation are continuing to emerge. Two more recent genome-wide studies have explored BMP-responsive genes: the modENCODE project used ChIP to map Mad binding sites in embryos (MacArthur *et al*. 2009) and a second study performed a genome-wide analysis of transcription factors that are known to have critical roles in BMP-dependent signaling during embryonic DV patterning (Deignan *et al*. 2016). Search engines to find binding sites in specific genes are available through the modENCODE website and through a number of other sites, which can be accessed through Flybase (Thurmond *et al*. 2019) and through REDfly, a regulatory element database (Rivera *et al*. 2019). A third study took a computational approach aimed at identifying genes coordinately regulated by BMP signaling in the nervous system (Vuilleumier *et al*. 2019). Smad-binding BMP-AEs were first predicted in the genome, and then, in vivo transgenic reporter lines were tested for their responsiveness to BMP signaling. Importantly, the authors showed that the predicted *BMP-AE* motif responds to Smad-mediated transcription not only in *Drosophila* but also in the vertebrate CNS. Other outstanding reviews of Smad-regulated transcriptional response are (Gaarenstroom and Hill 2014; David and Massague 2018).

Although most studies have focused on Smads as the transducers of BMP signals, some evidence suggests that Mad and Medea interact with other transcription factors in the absence of a BMP signal. The major evidence comes from studies in S2 cells, where coexpression of either *Mad^10^* or *Mad^12^* gave 2-fold elevated expression from a Wnt reporter with TCF-binding sites, compared its expression in the absence of Mad (Eivers *et al*. 2011). These authors proposed that unphosphorylated Mad takes part in a Mad–Pangolin–Armadillo complex and mediates responses to Wg signaling. Phenotypes from RNA interference knockdown of *mad* in embryos and wing disc clones exhibit *wg*-like phenotypes, supporting this proposal. Intriguingly, a Wg-like phenotype from overexpression of a specific *Medea* allele, *Medea^R100T^*, in the wing has been reported (Takaesu *et al*. 2008), but it remains unclear how this DNA-binding site mutant could impact the function of a Mad–Pangolin–Armadillo complex. Isolation of a molecular null allele for Mad would be useful to resolve the question of non-BMP signaling functions for Mad.

#### Posttranslational modification of Medea impacts gene expression

The dynamics of Medea nuclear import and export also are influenced by posttranslational modifications. Medea can be ubiquitylated as demonstrated directly for the ubiquitin ligase Highwire at postsynaptic NMJs (McCabe *et al*. 2004). Evidence for the role of ubiquitylation of Medea in embryonic and imaginal disc patterning comes from a requirement for the deubiquitylase Fat facets (Stinchfield *et al*. 2012). Maternally provided Fat facets deubiquitylase is necessary for full expression of the embryonic BMP target genes *rhomboid* and *hindsight* in the presence of wild-type Medea, but not when a transgenic nonubiquitylatable Medea mutant (Med^K738R^) is present. Evidence based on genetic interactions suggests that embryonic ubiquitylation of Medea is mediated by Nedd4 (Wisotzkey *et al*. 2014).

The dynamics of Medea accumulation in the nucleus is also influenced by sumoylation, which occurs in the nucleus. Components of the sumoylation pathway are maternally loaded into the embryo; the sumo E2 conjugating enzyme, Ubc9, is encoded by the *lesswright* gene, which is necessary to limit the spatial domains for expression of BMP target genes *race*, *hindsight*, *tailup*, and *u-shaped* (Miles *et al*. 2008). Medea is sumoylated on 3 lysines, K113, K159, and K222; nuclear export is greatly enhanced by sumoylation at all 3 sites.

Finally, mammalian Smad4 has proline-direct kinase phosphorylation sites within its linker domain, which prime for GSK3 phosphorylation (Bruce and Sapkota 2012; Demagny and De Robertis 2016) Similar sites can be found in Medea with online phosphosite searches, but their significance is unknown.

#### Smad interactions impact signaling output

In most tissues, BMP activity stimulates the expression of the *daughters against dpp* (*Dad*) gene, which encodes iSmad (Tsuneizumi *et al*. 1997). Dad interacts with the BMP type I receptors, Sax and Tkv, to downregulate their function (Inoue *et al*. 1998; Kamiya *et al*. 2008), providing a negative feedback mechanism that stabilizes signal levels and smooths fluctuations in responses from cell to cell across a tissue (Ogiso *et al*. 2011). While Mad and Smox/dSmad2 act in the 2 distinct, highly conserved BMP and Activin signal transduction pathways, their relative endogenous concentrations are keys to maintaining their distinct functions. Surprisingly, Mad can be phosphorylated by the *Drosophila* activin type I receptor Babo, a mechanism enhanced when Smox/dSmad2 levels are low (Peterson *et al*. 2012). Interactions between these Smad signaling systems can be detected when relative Smad levels are manipulated in wing disc development, either by reducing levels of expression for one Smad or by overexpression (Sander *et al*. 2010; Hevia and de Celis 2013; Peterson and O’Connor 2013; Kane *et al*. 2018). These observations provide a caveat to many, if not all studies of BMP signaling that involve manipulation of Mad levels by RNA interference knockdown or use of hypomorphic alleles. In this case, normal levels of Smox/dSmad2 allow it to out-compete Mad, for binding to activated Babo, but an imbalance in their relative concentrations creates permissive conditions for Mad activation by Babo. In such a situation, Activin-like signaling through Babo will activate BMP response genes.

##### Subcellular localization of pMad: Gbb signaling at the NMJ

A fruitful system for understanding the nuances of intracellular signaling comes from the specific role of the Gbb ligand as a retrograde signal controlling muscle to motor neuron communication at the larval NMJ. *gbb* is required for synaptic growth of the NMJ, and *gbb* mutants show a loss of pMad in the CNS of late embryos (McCabe *et al*. 2003). A better restoration of neuronal pMad is seen when *gbb* is expressed in muscles when compared with pan-neuronally, leading to the proposal that Gbb provides a retrograde signal. The function of the type II receptor *wit* (Aberle *et al*. 2002; Marqués *et al*. 2002) along with type I receptors *tkv* (Marques *et al*. 2003; Marques and Zhang 2006) and *sax* is required to receive the Gbb signal and to phosphorylate Mad presynaptically (Rawson *et al*. 2003). As discussed above, multiple forward genetic screens and reverse genetic studies have unveiled the critical importance of endocytosis and trafficking of active signaling complexes for fine-tuning signaling activity at the NMJ.

As discussed above, 3 types of BMP signaling have been revealed at the *Drosophila* larval NMJ: (1) canonical Gbb-induced Sax/Tkv/Wit mediates phosphorylation of Mad critical for synaptic growth, (2) local synaptic signaling involving phosphorylation of Mad by Sax/Tkv/Wit in a Gbb-independent manner, and (3) noncanonical Gbb signaling that influences LimK association with Wit, affecting actin dynamics (reviewed in Upadhyay *et al*. 2017; Vicidomini and Serpe 2022). For canonical signaling, the considerable physical separation of the NMJ from the nucleus requires that the intracellular signal (pMad) must be trafficked along the microtubules of the axon to the soma or cell body. Vesicles containing internalized Gbb/Wit/Tkv complexes are directly transported along the axonal microtubules to the cell body (Smith *et al*. 2012; Kang *et al*. 2014), and accumulation of pMad in the cell body depends on the microtubule motor protein Dynein and its cargo adaptor Dynactin (Eaton *et al*. 2002; McCabe *et al*. 2003). pMad and Medea accumulation in the nucleus regulate target gene expression, necessary for the expansion of neuronal arborization at the NMJ, in response to an ∼100 × growth in muscle size (Gorczyca *et al*. 1993; Keshishian *et al*. 1993). Active Gbb/Wit/Tkv complexes continue to phosphorylate Mad C-terminal serines (pMad) after the ligand–receptor complex is endocytosed, and this activity contributes significantly to overall levels of signaling, as measured by NMJ growth (Dickman *et al*. 2006; O’Connor-Giles *et al*. 2008). However, it seems likely that the active receptor complexes are not the only modulators of pMad levels in the cell body.

Partitioning of pMad between the synapse and the cell body is directly impacted by the kinase Nemo [*nmo* (Choi and Benzer 1994)] and activity of the ionotropic glutamate receptors [GluRIIA receptors (Sulkowski *et al*. 2014, 2016)]. Nemo phosphorylates Mad at S^25^, on the N-terminal side of the MH1 domain (Zeng *et al*. 2007). Mutants for *nmo* show increased pMad accumulation at NMJ synapses with an associated decrease of pMad levels in the cell bodies of the ventral nerve cord (Merino *et al*. 2009). In contrast, Nemo overexpression in motor neurons resulted in increased cell body accumulation of Myc-tagged Mad in conjunction with low nuclear accumulation. These data suggest that pS^25^Mad is preferentially transported from the synapse to the cell body but does not tend to accumulate in the nucleus. Consistent with these results, an early study of *nmo* mutant phenotypes in wing imaginal discs suggested that Nemo kinase antagonizes BMP signaling (Zeng *et al*. 2007). Surprisingly, the overexpression of Nemo in motor neurons had no impact on synaptic growth, suggesting that pMad resides in the nucleus long enough to normally regulate the necessary gene expression, even in the presence of excess Nemo activity. In contrast, Nemo overexpression reduced the strength of the synapse, as measured by neurotransmitter release (Sulkowski *et al*. 2014; 2016). Altogether, data support a model in which Nemo phosphorylation of Mad promotes the accumulation of C-terminally phosphorylated Mad at the presynaptic terminus, where it influences the maturation of the synapse. This accumulation is directly impacted by the activity of type A glutamate receptors at the synapse as discussed above. How pMad acts at the cell periphery remains an open question; some evidence suggests that the phosphorylated Mad continues to associate with the synaptic BMP receptor complex to modulate its ability to continue signaling (Vicidomini and Serpe 2022).

Interestingly, the dSod1^G85R^ knockin ALS model shows an elevation in synaptic pMad but no change in nuclear pMad in motor neuron cell bodies (Held *et al*. 2019). The motor dysfunction associated with dSod1^G85R^-ALS is alleviated by BMP signaling induced by the overexpression of Gbb or activated Sax, which alleviates motor dysfunction when expressed in a variety of neurons, both glutamatergic motor and cholinergic sensory neurons, but not in muscle or glia, indicating that activation of the pathway postsynaptically is not sufficient to restore motor function. Given that synaptic pMad appears to act as a sensor of synaptic activity but not synaptic growth, this finding would be consistent with the conclusion that dSod1G85R-ALS animals are hyperactive for synaptic function but unchanged for synaptic growth. Indeed, no significant change in NMJ area and bouton number was observed in A2 and A3 at NMJ4 and NMJ6/7 in dSod1^G85R^-ALS larvae (Held *et al*. 2019).

Together these studies highlight the strength of *Drosophila* genetics to probe the differential effects from altered Mad partitioning between distinct subcellular locations and emphasize the importance of in vivo studies to probe the nuances of BMP signal regulation.

## Mathematical models of BMP signaling

BMP signaling in different Drosophila tissue contexts has provided powerful examples that reinvigorated the discussion of morphogen gradient and tissue organizer theories (Wolpert 1969; Gurdon and Bourillot 2001; Tabata 2001; Wolpert 2010; Meinhardt 2015). Computational modeling has been applied in several of these contexts as a conceptual platform to generate a snapshot of the complexity of BMP signaling mechanisms. Modeling serves 3 important purposes: (1) it provides a framework to explain a large amount of data, (2) it can be used to assess the likelihood that specific parameters play a role in a proposed mechanism by comparison of the computational model with experimentally observed features, and (3) it can highlight inconsistencies in the interpretation of data, making predictions that can be tested empirically. Modeling has been deployed repeatedly to assess the impacts of factors that modulate the BMP activity gradient and its signaling responses or to identify unanswered questions about how the pathway might be regulated (some examples include: Mizutani *et al*. 2005; Lander *et al*. 2009; Lei and Song 2010; Umulis *et al*. 2010; Wartlick *et al*. 2011; Umulis and Othmer 2015; Chen 2019; Chen and Zou 2019; Zhu *et al*. 2020; Madamanchi *et al*. 2021). Specific examples for mechanisms discussed above include modeling to define parameters of Smad nuclear accumulation in mammalian cells, providing support for the prevailing Smad nuclear shuttling model (Schmierer *et al*. 2008), and modeling to formalize the proposed mechanism for Dpp/Scw heterodimer transport and release by Sog, Tsg, and Tld (Umulis *et al*. 2010). Studies of Drosophila BMP signaling gradients have also been used in discussions of critical issues for quantitative image analysis used as the basis for computational models (Brooks *et al*. 2012; Kicheva *et al*. 2013), for computational fitness testing (Pargett and Umulis 2013) of a given model and of the distinct constraints (Lander *et al*. 2009; Huang and Umulis 2018) and for different aspects of gradient modeling.

The nature of the BMP activity gradient in the wing disc has been an attractive subject to test theories for the establishment of patterned gene expression and control of organ growth. How the BMP activity gradient is formed has been one focus for modeling, with various mechanisms proposed to deploy ligands from a localized site of expression (Lander *et al*. 2009; Kicheva *et al*. 2013; Matsuda *et al*. 2016; Madamanchi *et al*. 2021; Stapornwongkul and Vincent 2021; Simsek and Özbudak 2022). The imaginal discs grow extensively during larval life, and their size increases by several orders of magnitude as patterning proceeds during the third larval instar (Curtiss *et al*. 2002). Scaling of the BMP morphogen gradient with wing primordium size has received significant attention, with several proposed mechanisms supported or tested by modeling (Teleman and Cohen 2000; Umulis 2009; Ben-Zvi *et al*. 2011; Hamaratoglu *et al*. 2011; Romanova-Michaelides *et al*. 2022).

Another area of intensive focus is related to observations that BMP signaling is required for imaginal disc growth as well as for patterning (Schwank and Basler 2010). This relationship is problematic and raises the question of how a graded distribution of BMP activity can give rise to uniform cell proliferation and growth (Milan *et al*. 1996). Various BMP-dependent mechanisms have been proposed and debated (Wartlick *et al*. 2011; Schwank *et al*. 2012; Averbukh *et al*. 2014; Restrepo *et al*. 2014; Romanova-Michaelides *et al*. 2015). Another view of growth regulation considers mechanisms that integrate chemical signaling from a BMP or Wg morphogen gradient with mechanical forces arising from cell packing and cell stretching within the epithelial primordium (Irvine and Shraiman 2017; Dye *et al*. 2021; Harmansa and Lecuit 2021). Such forces are commonly thought to be transmitted through adherens junctions, their associated actin-myosin network, and potentially by Hippo signaling, which is thought to respond to epithelial cell packing for control tissue growth control (Dupont 2016; Sun and Irvine 2016; Irvine and Shraiman 2017).

Our limited discussion of BMP gradient scaling with tissue growth scratches the surface of the signaling complexity that regulates patterning and growth. In addition to signaling cross talk between BMP and Hippo signaling, it is also clear that cross talk between the BMP and Activin pathways, BMP and Wg/Wnt pathways, and BMP and Receptor Tyrosine Kinase pathways, such as integrin, EGF, and FGF signaling, are critically important. To understand complex physiological systems, modeling may provide a means to frame the next research questions in this vein. As the field moves forward, future models will depend on specific observations, reagents, and measurements made empirically and should replicate what is observed in vivo. Its power lies in its ability to encompass the complexity of a system, spatially and temporally, and make predictions that can be tested in situ. Continuing the successful combination of computational and experimental analysis will advance the fields’ understanding of the context-dependent nature of a pathway like BMP signaling.

## Conclusions and future challenges

Over the past 30+ years, *Drosophila* research has been instrumental in (1) deciphering the components of BMP signaling and (2) elucidating a range of molecular mechanisms that regulate this multifunctional signaling pathway. *Drosophila* geneticists worked very effectively with other researchers using vertebrate systems in a symbiotic relationship to rapidly advance the BMP signaling field. This synergy established the core signaling pathway, created reagents, and opened the door for continued investigations. While the core signaling pathway is quite simple and made up of 3 functional components: the BMP signaling molecule or ligand, the transmembrane BMP receptors, and the Smad signal transducer, each component is a multimer comprised of related proteins. The individual proteins are evolutionarily conserved, exhibiting a degree of functional conservation. The complexity of this pathway, which enables its use in a multitude of diverse processes during development and in tissue homeostasis, is evident in many molecular mechanisms that regulate its signaling output or activity. The types of mechanisms act at every level of the pathway and range from transcriptional and translational regulation of each core component to posttranslational modifications, combinatorial formation of the multimers, processing, secretion/delivery to the cell surface, extracellular interacting proteins, ligand/receptor binding affinities, kinase activation, translocation into nucleus, associated transcriptional regulators, DNA-binding site affinities, trafficking, recycling, dephosphorylation/inactivation, and degradation.

In this review, we attempted to provide the reader with examples of the breadth of regulatory mechanisms based on experimental evidence. Due to the multimeric nature of BMP signaling components and thus the sensitivity of such a system to imbalances in the stoichiometry of respective elements, we have highlighted studies that were performed in vivo and/or investigated components expressed at endogenous levels. *Drosophila* have fewer BMP signaling components than vertebrates, and therefore, as a research organism, it may provide a system where the importance of stoichiometry can be best studied, especially given the relative ease with which genetic manipulations allow for in vivo analysis. Biochemical studies using vertebrates have been critical in determining macromolecular structures, binding affinities, and the formation of multimeric complexes such as homodimeric versus heterodimeric ligands and the composition of heterotetrameric receptor complexes. However, detecting and/or visualizing different complexes in vivo requires technical advances. Such developments will allow for a thorough understanding of how a relatively simple signaling pathway controls a multitude of cell–cell communication events. The *Drosophila* system has proven its value in pathway analysis and may be the most amenable to studying the functional requirements of different components, especially those variants that arise from alternative splicing and produce slightly different protein isoforms. Such variants have been documented, but more detailed research is required to fully appreciate their functional significance. Another area of importance that is especially relevant to the large classes of mammalian ligands and receptors is how are the proper ratios of homodimer and heterodimer ligands and receptor complexes established in a given tissue? The field has begun to uncover the existence of different pools of ligands and receptor complexes and seen that their presence varies from tissue to tissue. The genes encoding different ligand or receptor monomers must be expressed in the same cell, yet we know that simply being expressed does not ensure an active source of ligand or the reception of a signal. When cells produce a mixed population of ligands and/or receptors, what dictates the bioactive molecules? Selective or conditional manipulation of components in vivo contexts coupled with advances in optogenetics will aid progress in this respect. In a similar vein, under what contexts are both Mad and Medea engaged in facilitating a transcriptional response? How is that response altered by associated transcriptional regulators? Significant progress has been made in this area. However, developing new genetic tools such as an engineered *mad* null allele, *mad^KO^*, (Mosallaei 2021) and optogenetic tools that reveal different phosphorylated forms of Mad will significantly advance our understanding of the functional intersection of other pathways with BMP signaling.

Overall, the past 20 years of research on BMP signaling has shown us that there are multiple layers of regulation required to ensure precise signaling outputs. The universality of these layers of regulation across the evolutionary spectrum has been tested in some cases; however, much more comparative research is needed. These will be important to resolve questions of the nuances of BMP signaling that appear to be critically sensitive during both development and in disease: How is the duration of signaling activity output controlled? What roles do feedback and feedforward regulation play in regulating output in different contexts? How do different ligand–receptor complex affinities impact spatiotemporal signaling? What dictates which regulatory mechanism is employed in different contexts? Are there compensatory mechanisms at play? We anticipate that the next 20–30 years of BMP research will continue to benefit from the collaborations between *Drosophila* researchers and those using other models to explore this fascinating and essential signaling pathway.
